# Microglia preconditioned by oxygen-glucose deprivation promote functional recovery in ischemic rats

**DOI:** 10.1038/srep42582

**Published:** 2017-02-14

**Authors:** Masato Kanazawa, Minami Miura, Masafumi Toriyabe, Misaki Koyama, Masahiro Hatakeyama, Masanori Ishikawa, Takashi Nakajima, Osamu Onodera, Tetsuya Takahashi, Masatoyo Nishizawa, Takayoshi Shimohata

**Affiliations:** 1 Department of Neurology, Brain Research Institute, Niigata University, 1-757 Asahimachi-dori, Chuoku, Niigata, Japan; 2 Department of Neurology, Niigata National Hospital, National Hospital Organization, 3-52 Akasaka-cho, Kashiwazaki, Niigata, Japan

## Abstract

Cell-therapies that invoke pleiotropic mechanisms may facilitate functional recovery in stroke patients. We hypothesized that a cell therapy using microglia preconditioned by optimal oxygen-glucose deprivation (OGD) is a therapeutic strategy for ischemic stroke because optimal ischemia induces anti-inflammatory M2 microglia. We first delineated changes in angiogenesis and axonal outgrowth in the ischemic cortex using rats. We found that slight angiogenesis without axonal outgrowth were activated at the border area within the ischemic core from 7 to 14 days after ischemia. Next, we demonstrated that administration of primary microglia preconditioned by 18 hours of OGD at 7 days prompted functional recovery at 28 days after focal cerebral ischemia compared to control therapies by marked secretion of remodelling factors such as vascular endothelial growth factor, matrix metalloproteinase-9, and transforming growth factor-β polarized to M2 microglia *in vitro/vivo*. In conclusion, intravascular administration of M2 microglia preconditioned by optimal OGD may be a novel therapeutic strategy against ischemic stroke.

More than half of patients who survive ischemic stroke suffer from motor disabilities^1^. To date, physical rehabilitation has remained the only effective therapeutic option to prompt functional recovery in these patients^2^. It is necessary, therefore, to establish other therapeutic strategies to facilitate functional recovery in stroke patients in the subacute and chronic phases.

It has been demonstrated that angiogenesis is a necessary process for functional recovery after ischemia in the heart^3^, which raises the possibility that enhancement of angiogenesis is one strategy for facilitating functional recovery after ischemic stroke^4^. In fact, a previous study using a rodent model demonstrated that enhanced angiogenesis via the intravenous administration of vascular endothelial growth factor (VEGF) at 2 days after cerebral ischemia promoted functional recovery^5^. However, systemic injection of VEGF causes adverse effects, including hypotensive and tachycardic responses in various animals^6^,^7^,^8^. Furthermore, early administration of VEGF might prompt blood-brain barrier (BBB) disruption that causes cerebral oedema and haemorrhagic transformation^5^. Therefore, a therapeutic strategy that enhances angiogenesis without these adverse effects is desirable.

“Single-target” therapies may be insufficient because ischemic cerebral injury involves several mechanisms. It has been proposed that therapeutic approaches should target multiple cell types to enhance protection and recovery^9^. Cell therapies using bone marrow mononuclear cells or bone marrow-derived mesenchymal stem/stromal cells may be an effective “multi-target” therapeutic strategy to facilitate functional recovery in stroke patients in the subacute and chronic phases via pleiotropic mechanisms^9^,^10^. One such mechanism observed in cell-based therapies using neural progenitor cells, bone marrow stromal cells, and mesenchymal stem cells was the induction of angiogenesis via secretion of VEGF or brain-derived neurotrophic factor (BDNF)^11^,^12^,^13^. Enhancement of axonal outgrowth after such cell-based therapy also was reported^13^,^14^. However, a recent multi-centre randomized controlled trial demonstrated that there was no beneficial effect of intravenous administration of bone marrow mononuclear stem cells among patients with ischemic stroke^15^. In addition, there remain several clinical concerns, including the efficiency with which bone marrow-derived cells cross the BBB^16^ and the technical safety of bone marrow-derived cell collection in patients who are undergoing anticoagulant or antiplatelet therapy for secondary prevention of stroke.

Cell-based therapies using microglia might be a promising novel therapeutic strategy because glial cells are the main source of the above-mentioned growth factors in the CNS^9^,^17^. Although several studies have demonstrated that microglia might expand cerebral infarct volume in the acute phase^18^,^19^, microglia after cerebral ischemia in the subacute and chronic phases are known to play protective roles via tissue and vascular remodelling^19^,^20^. These ischemia-induced protective microglia are called M2 microglia, and their protective effect is considered to be due to the secretion of remodelling factors, such as VEGF and BDNF, matrix metalloproteinase-9 (MMP-9), and transforming growth factor-beta (TGF-β) after ischemia^17^,^20^,^21^,^22^,^23^,^24^,^25^, that may facilitate anti-inflammation, angiogenesis, and axonal outgrowth after cerebral ischemia. In addition, transplanted microglia can cross the BBB, particularly in the ischemic condition^26^,^27^,^28^. Therefore, we speculated that a cell-based therapy using microglia preconditioned by optimal ischemic condition might be an ideal and convenient therapeutic strategy for ischemic stroke.

In the present study, we hypothesized that microglia preconditioned by oxygen-glucose deprivation (OGD) and administered intra-arterially may cross the BBB, secrete remodelling factors in the brain parenchyma, and exert pleiotropic therapeutic effects via the promotion of angiogenesis and axonal outgrowth against focal cerebral ischemia even in the subacute phase. To test this hypothesis, we first delineated temporal changes in angiogenesis and axonal outgrowth in the ischemic core and penumbra in a rat model of transient focal cerebral ischemia. Then, we investigated whether primary microglia preconditioned by optimal OGD promoted functional recovery via enhancement of angiogenesis and axonal outgrowth after focal cerebral ischemia.

## Results

### Angiogenesis on the border of the ischemic core in the subacute phase of focal cerebral ischemia

**Figure 1:**
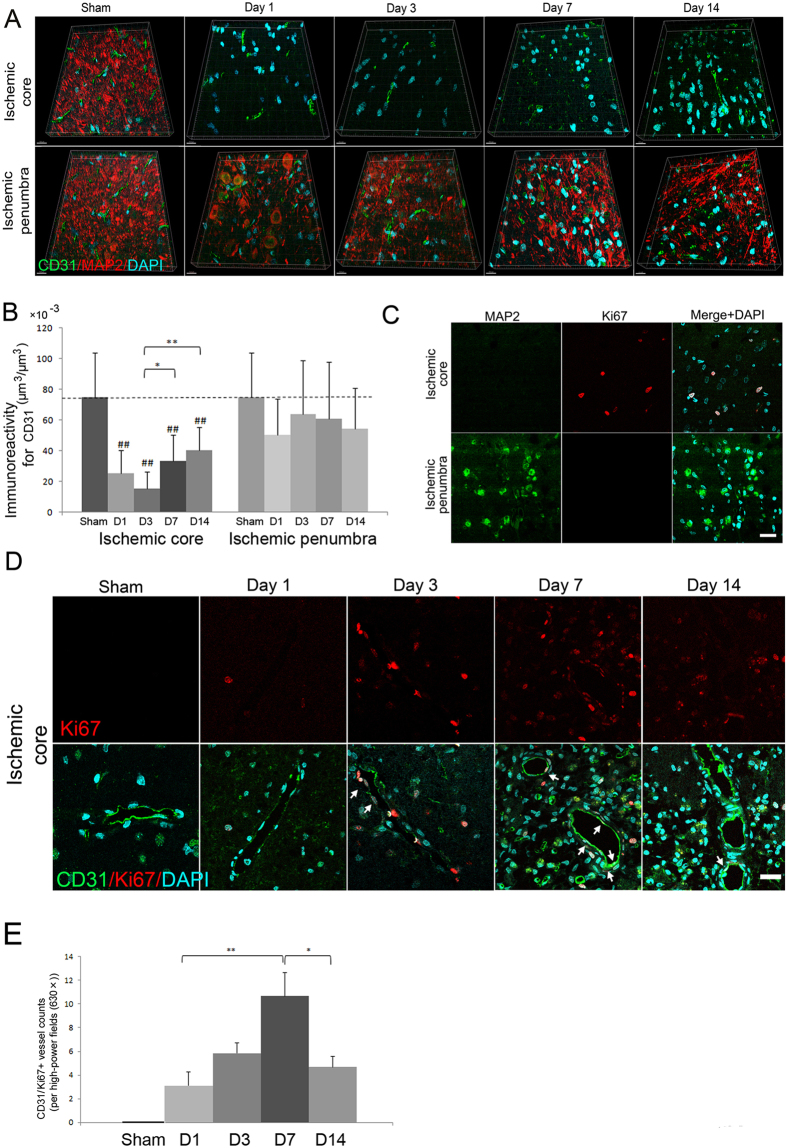
Temporal changes in angiogenesis in the rat cerebral cortex after focal cerebral ischemia. (**A**) Cluster of differentiation 31 (CD31; green)/microtubule-associated protein 2 (MAP2; red)/4′,6′-diamidino-2-phenylindole (DAPI; blue) triple labelling of cerebral cortices in the non-ischemic (sham-operated) and ischemic core and penumbra at 1, 3, 7, and 14 days after cerebral ischemia examined by confocal microscopy. CD31 and MAP2 are markers for angiogenesis and neuronal dendrites, respectively. Three-dimensional reconstruction images show temporal changes in angiogenesis and neuronal dendrites in the ischemic core and penumbra. A secondary-only antibody control confirms its specificity. Scale bars, 15 μm. (**B**) The immunoreactivity of CD31-positive volume (μm^3^) per unit volume (μm^3^) at 1 (D1), 3 (D3), 7 (D7), and 14 (D14) days after cerebral ischemia (N = 21). *P < 0.05, **P < 0.01, ^##^P < 0.01 versus sham-operated rats. (**C**) MAP2 (green)/Ki67 (red)/DAPI (blue) triple labelling of cerebral cortices in the ischemic core and penumbra examined by confocal microscopy. Ki67 is a marker for cellular proliferation at 7 days after cerebral ischemia. Scale bars, 20 μm. (**D**) CD31 (green)/Ki67 (red)/DAPI (blue) triple labelling of cerebral cortices in the non-ischemic (sham-operated) and ischemic core at 1, 3, 7, and 14 days after cerebral ischemia examined by confocal microscopy. Arrows indicate Ki67-positive angiogenic endothelial nuclei. Scale bars, 20 μm. (**E**) The numbers of CD31/Ki67 double-positive vessels from the ischemic core at 1 (D1), 3 (D3), 7 (D7), and 14 (D14) days after cerebral ischemia (N = 21). *P < 0.05, **P < 0.01.

To determine the detailed localization and temporal changes of angiogenesis after transient focal cerebral ischemia, we performed immunofluorescence staining against the ischemic cortex of Sprague-Dawley rats using an antibody against the endothelial and angiogenesis marker, cluster of differentiation (CD)31 (Fig. 1A and B). Confocal microscopic studies revealed that the immunoreactivity for CD31 volume per unit volume on the border area within the ischemic core defined as microtubule-associated protein 2 (MAP2)-negative area decreased at 1 day after cerebral ischemia and reached a minimum level at 3 days after cerebral ischemia compared to sham-operated rats (all P < 0.001) (Fig. 1B). However, immunoreactivity for CD31 volume per unit volume in the same area was increased at 7 and 14 days after cerebral ischemia compared to that of 3 days after cerebral ischemia (P = 0.013 and P < 0.001, respectively). In contrast, no significant changes in the immunoreactivity for CD31 volume per unit volume were observed compared to sham-operated rats in the ischemic penumbra defined as MAP2-positive region (P = 0.083) (Fig. 1B).

We next investigated which cells were involved in angiogenesis after cerebral ischemia. We found that Ki67 was expressed in the border area within the ischemic core, but not in the ischemic penumbra or in sham-operated rats (Fig. 1C). Additionally, Ki67-positive cells on the border area within the ischemic core were slightly increased at 3 days and markedly increased at 7 days after cerebral ischemia (Fig. 1D and E). Ki67 expression was observed in the nuclei of endothelial cells, pericytes, and microglia but not in the nuclei of astrocytes (Fig. 1D, Supplementary Fig. 1). Ki67-positive endothelial cells on the border area within the ischemic core also were markedly increased at 7 days after cerebral ischemia in a rat autologous thromboembolic model with tissue plasminogen activator (Supplementary Fig. 2).

### No axonal outgrowth in the ischemic core and penumbra until 14 days after focal cerebral ischemia

**Figure 2:**
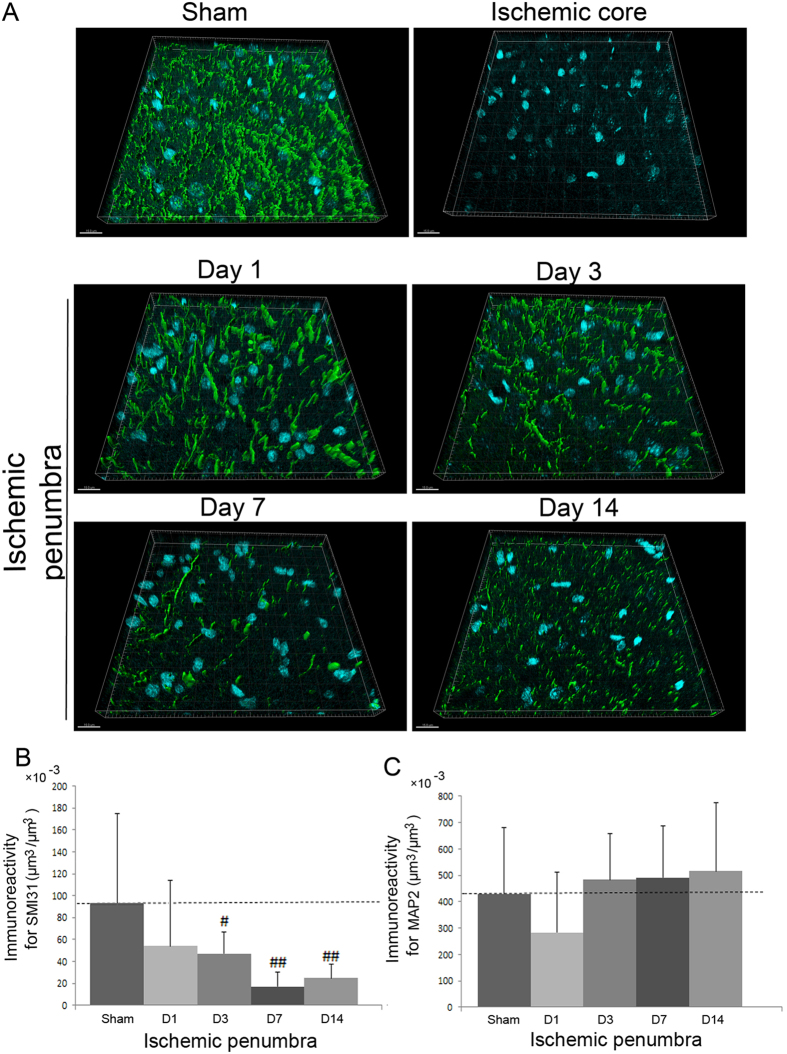
Temporal changes in neuronal axons and dendrites in the rat cerebral cortex after focal cerebral ischemia. (**A**) SMI31 (green)/4′,6′-diamidino-2-phenylindole (DAPI; blue) double labelling in the non-ischemic (sham-operated), ischemic core, and ischemic penumbra cortices at 1, 3, 7, and 14 days after cerebral ischemia examined by confocal microscopy. SMI31 is a marker for neuronal axons. A secondary-only antibody control confirms its specificity. Scale bars, 15 μm. **(B)** The immunoreactivity for SMI31 volume (μm^3^) per unit volume (μm^3^) in the ischemic penumbra at 1 (D1), 3 (D3), 7 (D7), and 14 (D14) days after cerebral ischemia (N = 21). ^#^P < 0.05 and ^##^P < 0.01 versus sham-operated rats. **(C)** The immunoreactivity for MAP2 volume in the ischemic penumbra (μm^3^) per unit volume (μm^3^) at 1 (D1), 3 (D3), 7 (D7), and 14 (D14) days after cerebral ischemia (N = 21).

To investigate neuronal regeneration after focal cerebral ischemia in the ischemic cortex of Sprague-Dawley rats, we performed immunofluorescence staining using antibodies against an axonal marker, SMI31 and a dendrite marker, MAP2, respectively^29^. Confocal microscopic studies revealed that there was no immunostaining of SMI31 and MAP2 in the ischemic core (Figs 1A and 2A). Furthermore, the immunoreactivity for SMI31 per unit volume on the border area within the ischemic penumbra decreased gradually after cerebral ischemia, and was not recovered even at 14 days after cerebral ischemia (day 3, P = 0.018; day 7, P < 0.001; day 14, P < 0.001) (Fig. 2A and B). In contrast, a transient decrease in the immunoreactivity for MAP2 per unit volume was observed only at 1 day after cerebral ischemia (P = 0.06) (Figs 1A and 2C).

### Therapeutic effects of microglia preconditioned by OGD against focal cerebral ischemia

**Figure 3:**
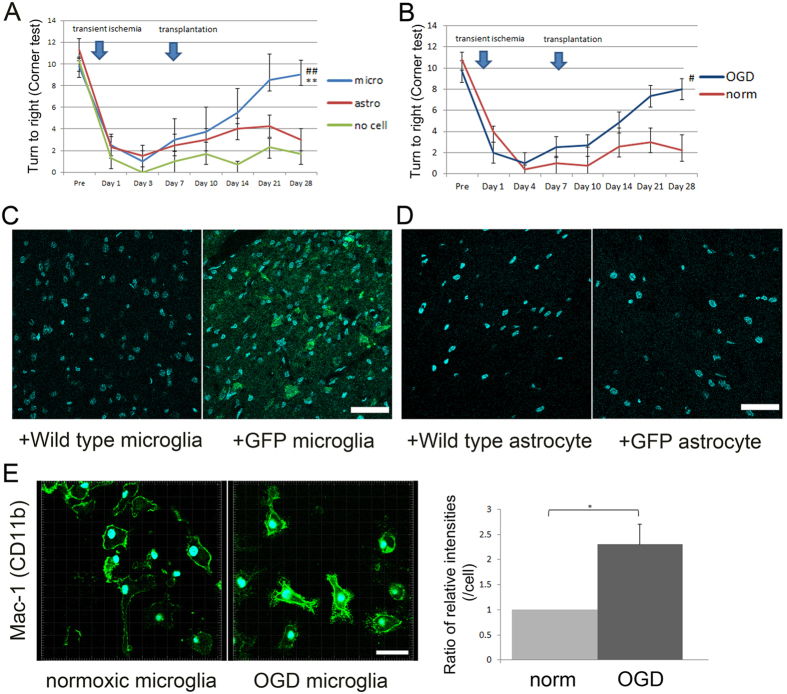
Improvements in neurological outcomes after oxygen-glucose deprivation (OGD)-preconditioned microglial transplantation. (**A**) Significantly better functional recovery on the corner test (performed 20 times) is observed in the OGD-preconditioned microglial transplantation group (micro group) compared with the vehicle-injected control group (no cell control group) and OGD-preconditioned astrocytic transplantation group (astro group); ^##^P < 0.01 vs astro group, **P < 0.01 vs no cell control group (N = 4 per group). (**B**) Significantly better functional recovery on the corner test (performed 20 times) is observed in the OGD-preconditioned microglial transplantation group compared with the normoxic microglial transplantation group (norm); ^#^P < 0.05 (N = 6). OGD-preconditioned microglia (**C**), but not OGD-preconditioned astrocytes (**D**), from GFP mice (green) migrate into the border area between the ischemic core and penumbra compared with those from wild-type mice at 3 days after transplantation. Scale bar, 50 μm. (**E**) Representative figures and bar graphs showing the relative signal intensities of adhesion receptor macrophage-1 antigen (Mac-1) (CD11b/CD18; green) from primary microglia under normoxic and OGD conditions. Confocal microscopic images of Mac-1 (green)/DAPI (blue) double labelling of microglia. White scale bars, 15 μm. The bar graph depicts the relative ratio of the signal intensities per cell of the normoxic samples to that of OGD-preconditioned samples (N = 5). A secondary-only antibody control was used to confirm specificity. *P < 0.05.

To investigate the effects of transplantation of primary microglia preconditioned by OGD against focal cerebral ischemia, we compared neurological outcomes among the no cell control group, OGD-preconditioned primary astrocytic transplantation group, and OGD-preconditioned microglial transplantation group by a sensorimotor scale after focal cerebral ischemia. Neurological deficit as measured by the corner test was significantly improved in the OGD-preconditioned microglial transplantation group compared to both the no cell control group and OGD-preconditioned astrocytic transplantation group at 28 days after cerebral ischemia (P = 0.006 and 0.024, respectively) (Fig. 3A). There were no significant differences in body weight at 1, 3, 7, 14, 21, and 28 days after cerebral ischemia among the three groups (P = 0.281) (Supplementary Fig. 3A).

Next, to investigate the effects of OGD preconditioning on functional recovery by microglial transplantation, we analysed neurological outcomes between the OGD-preconditioned microglial transplantation group and normoxia-microglial transplantation group by a sensorimotor scale after focal cerebral ischemia. Neurological deficit as measured by the corner test was significantly improved in the OGD-preconditioned microglial transplantation group compared with the normoxia-microglial transplantation group at 28 days after cerebral ischemia (P = 0.023) (Fig. 3B). There were no significant differences in body weight at 1, 7, 14, 21, and 28 days after cerebral ischemia between the groups (P = 0.71) (Supplementary Fig. 3B). We did not observe any allergic reactions or symptoms of graft versus host diseases after transplantation.

To confirm whether microglia and astrocytes preconditioned by OGD migrated into the brain parenchyma across the BBB, we administered both OGD-preconditioned primary microglia and astrocytes derived from green fluorescent protein (GFP) transgenic mice^28^,^30^. After intra-arterial administration of these cells, OGD-preconditioned GFP microglia were observed in the border area between the ischemic core and penumbra at 3 days after transplantation (Fig. 3C) but not at 21 days after transplantation. In contrast, no OGD-preconditioned GFP astrocytes were observed at either 3 or 21 days after transplantation (Fig. 3D). The leukocyte adhesion receptor macrophage-1 antigen (Mac-1) (CD11b/CD18) is a β2 integrin that is constitutively expressed on the surface of leukocytes and microglia, as well as quantitatively upregulated by inflammatory mediators and ischemia. Mac-1 mediates the firm adhesion of leukocytes to the blood vessel by binding to its endothelial ligand, intercellular adhesion molecule-1 (ICAM-1)/CD31. Deficiency in Mac-1 might decrease susceptibility to infiltration and migration after cerebral ischemia^31^,^32^. To investigate the effect of preconditioning by OGD on microglia, we compared the levels of Mac-1 expression on microglia under normoxic and OGD conditions. We found that the levels of Mac-1 after OGD preconditioning were twice those under normoxic conditions (P = 0.038) (Fig. 3E).

### Marked changes in levels of growth factors and MMP-9 secreted by OGD-preconditioned glial cells

To investigate the effect of preconditioning by OGD on microglia, we compared levels of VEGF and BDNF in microglial and astrocytic-conditioned media under normoxic and OGD conditions. As described in the Methods section, we previously determined that the optimal incubation time under the OGD condition was 18 h, because this condition induced sufficient oxidative and hypoglycaemic stress without causing cell death^33^,^34^. Additionally, M2 microglia after cerebral ischemia were detectable starting from 12 h, maximally increased at 24 h, and markedly decreased at later time points^20^. Therefore, we incubated microglia under OGD conditions for 18 h before transplantation.

**Figure 4:**
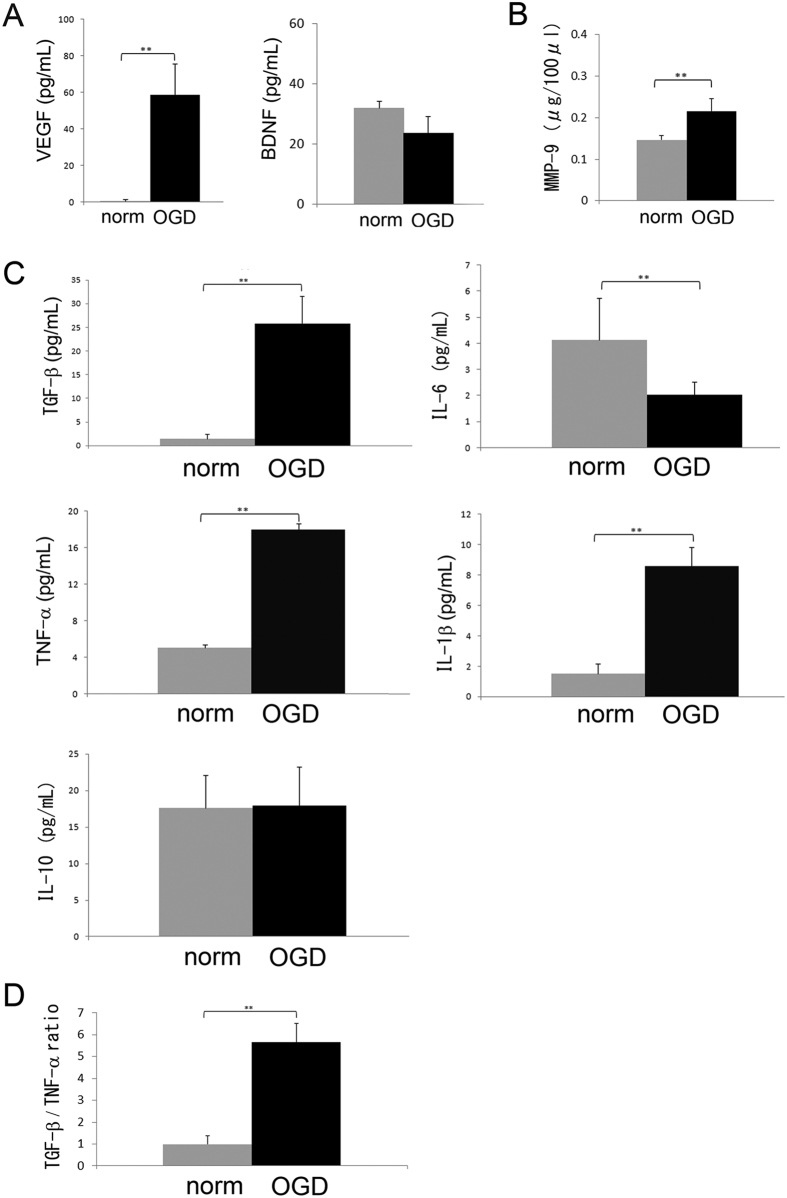
Characteristics of murine primary-cultured microglia preconditioned by oxygen-glucose deprivation (OGD). (**A**,**B**) The levels of secretory vascular endothelial growth factor (VEGF), brain derived neurotrophic factor (BDNF) (**A**) and matrix metalloproteinase-9 (MMP-9) (**B**) from conditioned media of murine primary-cultured microglia subjected to normoxia (norm) or OGD (N = 6–8 each). **P < 0.01. (**C**) The levels of secretory anti-inflammatory cytokines such as transforming growth factor-β (TGF-β) and interleukin-10 (IL-10), and pro-inflammatory cytokines such as IL-6, tumour necrosis factor-α (TNF-α), and IL-1β from conditioned media of murine primary-cultured microglia subjected to normoxia (norm) or OGD (N = 6 each). **P < 0.01. (**D**) The ratio of TGF-β per TNF-α, which reflects microglial polarization after normoxia and OGD preconditioning (N = 6 each). The increase in the ratio of TGF-β per TNF-α reveals the polarization of M2 microglia after OGD preconditioning. **P < 0.01.

We found that the levels of VEGF from microglial and astrocytic-conditioned media after OGD preconditioning were markedly higher than those under the normoxic condition (P = 0.006 and <0.001, respectively) (Fig. 4A, Supplementary Fig. 4A). In addition, the levels of BDNF from astrocytic-conditioned media but not microglial-conditioned media after OGD preconditioning were higher than those under the normoxic condition (P = 0.004 and P = 0.198, respectively) (Fig. 4A, Supplementary Fig. 4B).

We also measured the levels of MMP-9 in microglial and astrocytic-conditioned media after OGD preconditioning, because sources of MMP-9 after cerebral ischemia include microglia^35^ and potentially astrocytes^36^. We found that the levels of MMP-9 from microglial and astrocytic-conditioned media after OGD preconditioning were higher than those under the normoxic condition (P = 0.001 and 0.005, respectively) (Fig. 4B, Supplementary Fig. 4C).

Finally, to determine changes in the cytokine profiles of microglia preconditioned by OGD, we compared levels of several cytokines from microglia under normoxic and OGD conditions. Generally, M2 microglia secretes interleukin (IL)-10 and TGF-β, whereas M1 microglia secretes tumour necrosis factor alpha (TNF-α), IL-1β, and IL-6^19^,^20^. We found that the levels of anti-inflammatory cytokine TGF-β after OGD preconditioning were 25 times as high as those under the normoxic condition (P = 0.002), and that the levels of pro-inflammatory cytokine IL-6 after OGD preconditioning were half as much as those under the normoxic condition (P < 0.001). In contrast, pro-inflammatory cytokines TNF-α and IL-1β after OGD preconditioning were three and four times as high as those under the normoxic condition (P < 0.001 and P < 0.001, respectively), whereas there was no difference in the level of anti-inflammatory cytokine IL-10 from microglia between the OGD-preconditioning and normoxic conditions (P = 0.35) (Fig. 4C). The ratio of TGF-β per TNF-α, which reflects the polarization of M1 and M2 microglia^3^, from microglia preconditioned by OGD, was six times as high as that from microglia under the normoxic condition (P = 0.009) (Fig. 4D). Taken together, these results demonstrate that preconditioning by the optimal OGD condition can prime microglia into the anti-inflammatory M2 dominant subtype.

### Microglial M2 switch after intra-arterial administration of 18 h-OGD-preconditioned microglia

**Figure 5:**
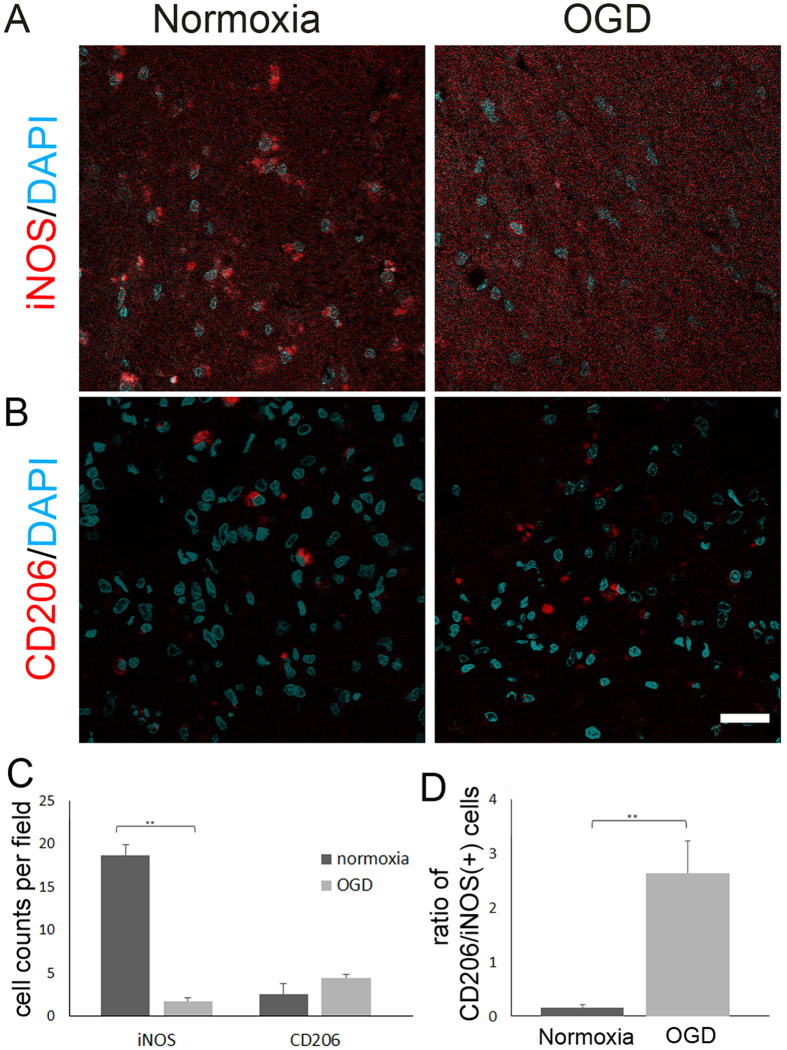
Transplantation of microglia preconditioned with oxygen-glucose deprivation (OGD) primes M2 microglial switch in the border area between the ischemic core and the penumbra at 3 days after transplantation. Representative figures indicate the number of cells positive for M1-specific marker (inducible nitric oxide synthase, iNOS) (**A**) and M2-specific marker (cluster of differentiation 206, CD206) (**B**) in the border area between ischemic core and penumbra from cerebral cortices of the normoxic microglial transplantation group or the OGD-preconditioned microglial transplantation group at 3 days after transplantation (10 days after cerebral ischemia). Confocal microscopic images showing iNOS or CD206 (red)/4′,6′-diamidino-2-phenylindole (DAPI; blue) double labelling in the ischemic cortices. Scale bars, 15 μm. (**C**) Bar graphs depicting the number of iNOS- and CD206-positive cells in the border between ischemic core and penumbra from cerebral cortices of the normoxic microglial transplantation group or OGD-preconditioned microglial transplantation group at 3 days after transplantation. (**D**) Bar graphs depicting the ratio CD206-positive cells to iNOS-positive cells, which reflects microglial polarization after normoxia and OGD preconditioning (N > 21). **P < 0.01.

To investigate the effect of OGD-preconditioned microglial transplantation on the injured brain parenchyma, we performed immunohistochemical analyses of the brains of transplanted rats at 3 days after transplantation using antibodies against inducible nitric oxide synthase (iNOS, M1 marker) (Fig. 5A) and CD206 (M2 marker) (Fig. 5B). Confocal microscopic examination revealed that the number of iNOS-positive cells in the border area between the ischemic core and the penumbra at 3 days after transplantation in the OGD-preconditioned microglial transplantation group was less than that in the normoxic microglia transplantation group (P < 0.001) (Fig. 5A and C). In contrast, the number of CD206-positive cells in the border area between the ischemic core and the penumbra at 3 days after transplantation in the OGD-preconditioned microglial transplantation group was more than that in the normoxic microglia transplantation group (P = 0.13) (Fig. 5B and C). The ratio of CD206-positive cells to iNOS-positive cells, which reflects the polarization of M1 and M2 microglia^19^, in the OGD condition was twenty times that in the normoxic condition (P = 0.001) (Fig. 5D). Taken together, these results demonstrate that OGD-preconditioned microglial transplantation can prime microglial M2 switch.

### VEGF, MMP-9, and TGF-β expression by OGD-preconditioned microglial transplantation

**Figure 6:**
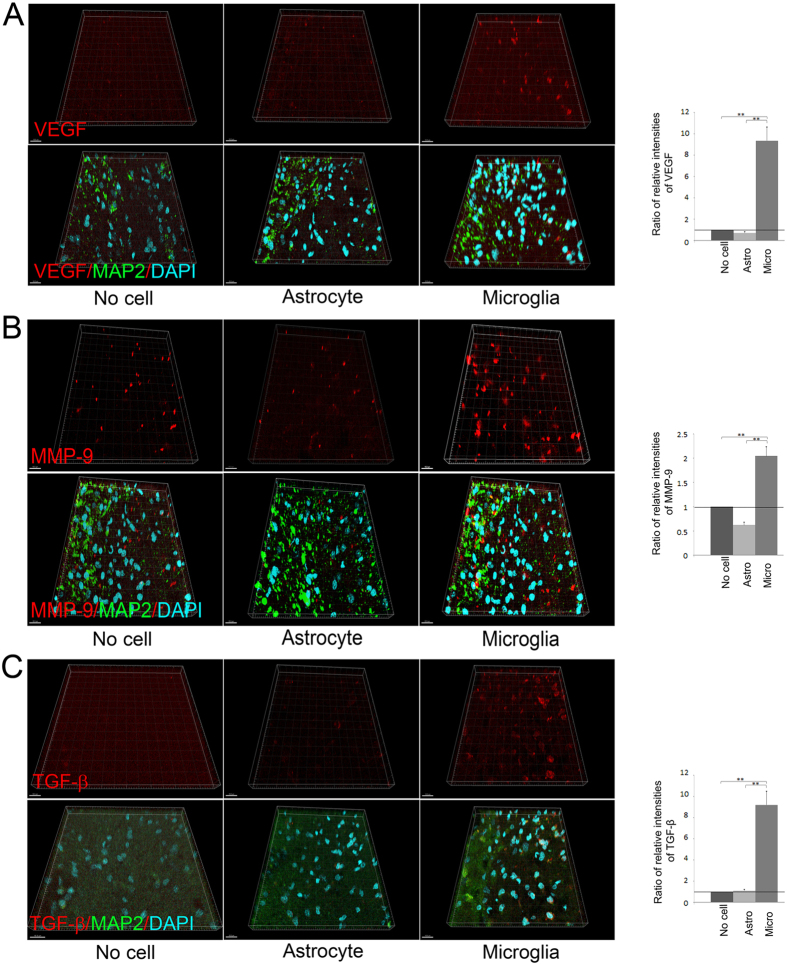
Transplantation of OGD-preconditioned microglia promotes expression of remodelling factors at 28 days after cerebral ischemia. (**A–C**) Representative figures and the relative signal intensities of vascular endothelial growth factor (VEGF) (**A**), matrix metalloproteinase-9 (MMP-9) (**B**), and transforming growth factor-β (TGF-β) (**C**) from cerebral cortices of the no cell control group and the microglia or astrocyte transplanted groups at 28 days after cerebral ischemia. VEGF (**A**), MMP-9 (**B**), and TGF-β (**C**) (red)/MAP2 (green)/DAPI (blue) triple labelling of cerebral cortices in the border between the ischemic core and penumbra at 28 days after reperfusion as examined by confocal microscopy. A secondary-only antibody control confirms its specificity. Scale bars, 15 μm. Bar graphs represent relative signal intensities of ischemic brain samples compared with those of no cell control samples (N = 21–28). **P < 0.01.

To confirm whether functional recovery after transplantation of microglia preconditioned by OGD was associated with upregulation of VEGF, MMP-9, and TGF-β in the injured brain parenchyma, we performed immunohistochemical analyses of the brains of transplanted rats using antibodies against VEGF, MMP-9, and TGF-β at 28 days after cerebral ischemia. Whereas expressions of VEGF, MMP-9, and TGF**-**β were undetectable in the brains of sham-operated rats, significant expressions of VEGF, MMP-9, and TGF-β were observed in the border area within the ischemic core and penumbra at 28 days after cerebral ischemia (21 days after transplantation). Analyses of immunoreactivity intensities demonstrated that the expressions of these remodelling factors were more prominent in the OGD-preconditioned microglial transplantation group than in the no cell control group and the OGD-preconditioned astrocytic transplantation group (both P < 0.001) (Fig. 6A–C). VEGF and MMP-9 expressions were observed not only in the microglia but also in pericytes, endothelial cells, and neurons within the ischemic cortex (Supplementary Figs 5 and 6). In addition, TGF-β expression was observed in microglia as well as pericytes and neurons within the ischemic cortex (Supplementary Fig. 7).

### Promotion of angiogenesis by OGD-preconditioned microglial transplantation

**Figure 7:**
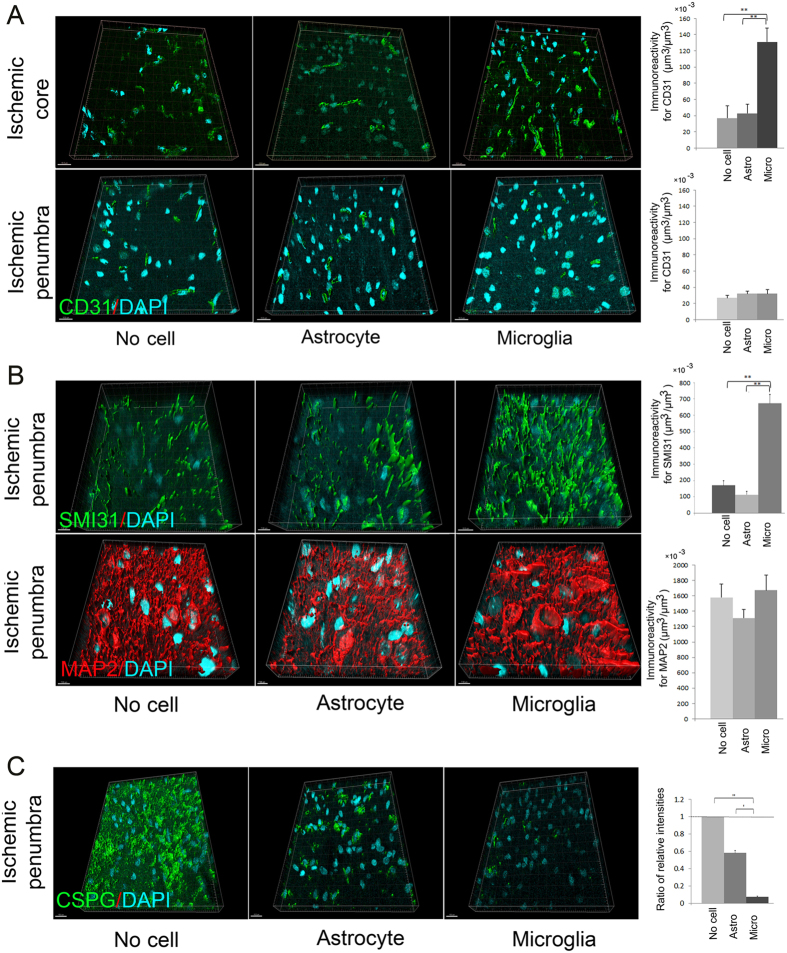
Transplantation of oxygen-glucose deprivation (OGD)-preconditioned microglia promotes angiogenesis in the border area within the ischemic core and axonal outgrowth in the ischemic penumbra at 28 days after cerebral ischemia. (**A**) Representative figures and bar graphs representing immunoreactivity for CD31 volume per unit volume, expressed as μm^3^ per μm^3^, in the ischemic core and penumbra from cerebral cortices of the no cell control group, OGD-astrocyte, or OGD-microglia transplanted groups at 28 days after cerebral ischemia. The cluster of differentiation (CD31; green)/4′,6′-diamidino-2-phenylindole (DAPI; blue) double labelling in the ischemic cortices at 28 days after cerebral ischemia as examined by confocal microscopy. Scale bars, 15 μm. (**B**) Representative figures and bar graphs representing immunoreactivity for SMI31 and MAP2-positive volumes per unit volume, expressed as μm^3^ per μm^3^, in the ischemic penumbra from the cerebral cortices of the no cell control group and OGD-preconditioned astrocyte or OGD-preconditioned microglia transplanted groups at 28 days after cerebral ischemia. SMI31 (green) or microtubule-associated protein 2 (MAP2; red)/DAPI (blue) double labelling in the ischemic cortices at 28 days after cerebral ischemia as examined by confocal microscopy. Scale bars, 7 μm. (**C**) Representative figures and bar graphs representing the relative signal intensities of chondroitin sulphate proteoglycan/neuron-glial antigen 2 (CSPG/NG2; green) from the cerebral cortices of the no cell control group and the OGD-preconditioned astrocyte or OGD-preconditioned microglia transplanted groups at 28 days after cerebral ischemia. CSPG/NG2 (green)/DAPI (blue) double labelling of the cerebral cortices in the ischemic penumbra at 28 days after cerebral ischemia as examined by confocal microscopy. Scale bars, 15 μm. The graph represents the relative signal intensities of the microglia or astrocytes transplanted into ischemic brain samples compared with samples from the no cell control group (N = 21–28). The bar graph represents the relative ratio of the signal intensities of the ischemic brain samples compared with that of sham-operated rat samples (N = 21–28). Moreover, a secondary-only antibody control confirms its specificity. *P < 0.05, **P < 0.01.

We speculated that expressions of VEGF, MMP-9, and TGF-β by OGD-preconditioned microglial transplantation might promote angiogenesis. Thus, we investigated the effects of OGD-preconditioned microglial transplantation on angiogenesis by immunofluorescence staining of the ischemic cortex at 28 days after cerebral ischemia using an antibody against the angiogenesis marker, CD31 (Fig. 7A). Confocal microscopic studies revealed that immunoreactivity for CD31 per unit volume in the border area within the ischemic core in the OGD-microglial transplantation group was more prominent than that in the no cell control group and OGD-preconditioned astrocytic transplantation group at 28 days after cerebral ischemia (at 21 days after transplantation) (P < 0.001 and P < 0.001). However, there was no significant difference in the immunoreactivity for CD31 per unit volume within the ischemic penumbra among the three groups.

### Promotion of axonal outgrowth by OGD-preconditioned microglial transplantation

We investigated the effects of OGD-preconditioned microglial transplantation on axonal outgrowth by immunofluorescence staining of the ischemic cortex at 28 days after cerebral ischemia using an antibody against the neurofilament protein marker, SMI31. The expression of SMI31 in the ischemic penumbra in the OGD-preconditioned microglial transplantation group was more prominent than that in the no cell control group and OGD-preconditioned astrocytic transplantation group (P < 0.001 and P < 0.001, respectively) (Fig. 7B). Additionally, the expression of another axonal outgrowth marker, growth associated protein 43 (GAP43)^14^, in the ischemic penumbra in the OGD-preconditioned microglial transplantation group also was more prominent than that in the no cell control group and OGD-preconditioned astrocytic transplantation group (P < 0.001 and P < 0.001, respectively) (Supplementary Fig. 8). In contrast, there was no significant difference in the expressions of MAP2 in the ischemic penumbra among the three groups (Fig. 7B).

To determine the mechanism by which OGD-preconditioned microglial transplantation promotes axonal outgrowth, we evaluated the expression of chondroitin sulphate proteoglycan (CSPG), because it inhibits axonal outgrowth^37^ and is cleaved and degraded by MMP-9^38^. To confirm our hypothesis that an increase in MMP-9 expression by OGD-preconditioned microglial transplantation may cause degradation of CSPG, resulting in axonal outgrowth, we performed immunofluorescence staining using an anti-CSPG/neuron-glial antigen 2 (NG2) antibody (NG2 is a major component of CSPG) (Fig. 7C). We compared intensities of CSPG/NG2 expression in sham-operated rats and transplanted rats at 28 days after cerebral ischemia. Confocal microscopic studies revealed that expression of CSPG/NG2 in the ischemic penumbra of the OGD-preconditioned microglial transplantation group was much lower than that in the no cell control group (P < 0.001) and OGD-preconditioned astrocytic transplantation group (P = 0.038) at 21 days after transplantation (at 28 days after cerebral ischemia).

## Discussion

**Figure 8:**
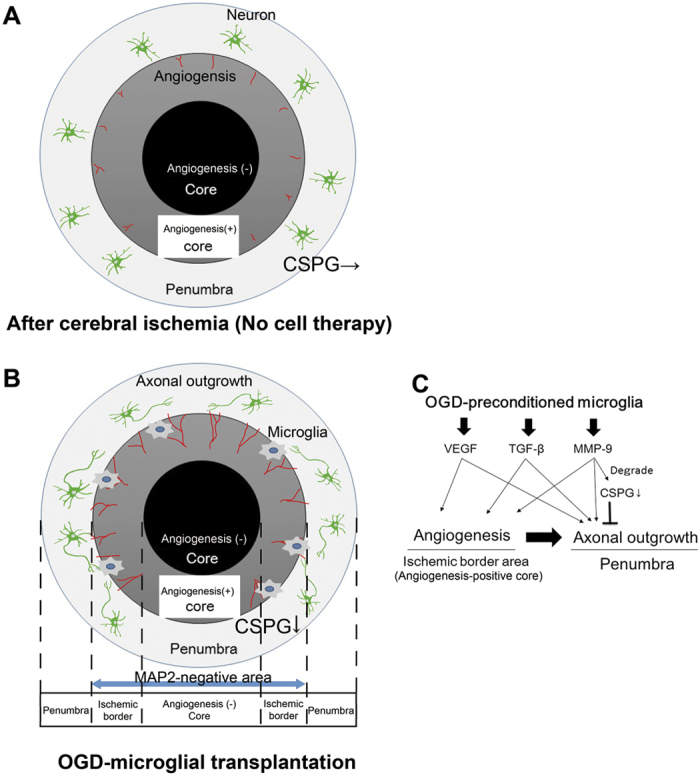
Mechanism of oxygen-glucose deprivation (OGD)-preconditioned microglial transplantation after cerebral ischemia. (**A**) The schema of angiogenesis after cerebral ischemia. The ischemic core is defined as the MAP2-immunonegative ischemic cortex. Angiogenesis (red lines) is slightly activated at the border area within the ischemic core, which we define as the “angiogenesis-positive core” (dark grey), by cell therapy. Thus, the MAP2-negative ischemic core consists of the angiogenesis-positive core and angiogenesis-negative core (black), which develops irreversible change. (**B**) The schema of angiogenesis and axonal outgrowth (regeneration, green cells; neurons) by OGD-preconditioned microglial transplantation (blue cells) after cerebral ischemia. OGD-preconditioned microglial transplantation markedly activated angiogenesis (red lines) at the angiogenesis-positive core (ischemic border area). In addition, the decrease in the expression of chondroitin sulphate proteoglycan (CSPG, grey and diagonal area), which is known to be an axonal outgrowth inhibitor, was observed in the ischemic penumbra after cerebral ischemia. An axonal outgrowth of neuronal cells (green) by OGD-preconditioned microglial transplantation was observed in the ischemic penumbra. (**C**) A diagram of therapeutic effects of OGD-preconditioned microglial transplantation after cerebral ischemia. Transplanted OGD-preconditioned microglia directly secreted vascular endothelial growth factor (VEGF), transforming growth factor-β (TGF-β), and matrix metalloproteinase-9 (MMP-9). These factors may also be associated with changes in resident native endothelial cells, astrocytes, pericytes, neurons, and microglia caused by the paracrine action of OGD-preconditioned M2 microglia. These factors directly prompt angiogenesis in the angiogenesis-positive core (ischemic border area). MMP-9 from microglia might degrade the axonal outgrowth inhibitor CSPG. Angiogenesis might facilitate the induction of axonal outgrowth. In addition, VEGF, TGF-β, and MMP-9 also might directly induce axonal outgrowth.

First, we investigated both temporal changes and the detailed location of angiogenesis and axonal outgrowth (neuronal regeneration) after cerebral ischemia, because angiogenesis and axonal outgrowth in the peri-infarct area were considered to promote functional recovery^13^,^39^. We demonstrated that endothelial proliferation and subsequent angiogenesis were activated at the border area within the MAP2-negative ischemic core but not in the ischemic penumbra from 7 days after cerebral ischemia. We confirmed these findings using a different ischemic model. Although there are several definitions of the ischemic core, the ischemic core in animal models is generally defined as the MAP2-negative lesion^40^,^41^. However, the present study demonstrated for the first time that the MAP2-negative ischemic core is not homogenous; angiogenesis was observed only in the border area within the MAP2-negative area (Fig. 8A). We speculated that the border area within the MAP2-negative ischemic core might be a novel therapeutic target area against cerebral ischemia. In contrast, axonal outgrowth was not observed in either the ischemic core or penumbra until 14 days after cerebral ischemia. This result was consistent with a previous report that axonal outgrowth was not observed until 28 days after cerebral ischemia^42^. These findings indicate that it could be difficult to promote axonal outgrowth, which is a prerequisite for functional recovery, without any sort of interventions after cerebral ischemia.

We demonstrated evident functional recovery by OGD-preconditioned microglial transplantation but not by OGD-preconditioned astrocytic transplantation. We also found that OGD-preconditioned microglia but not OGD-preconditioned astrocytes could cross the BBB to reach the injured brain parenchyma. This finding was consistent with several reports showing that primary microglia could cross the BBB^27^ and reach the injured brain parenchyma^22^,^26^,^28^. As Mac-1 mediates leukocyte and microglial adhesion to the endothelial surface, it might be important for the infiltration of these cells into the affected brain parenchyma^31^,^32^. Increasing the levels of Mac-1 after OGD preconditioning may enable the microglia to cross BBB and reach the injured brain parenchyma. Several studies have demonstrated that the chemokine stromal-derived factor-1 (SDF-1, also known as CXCL12) plays an important role in the homing of bone marrow-derived cells, especially microglia, monocytes, and stem cells, to areas of ischemic injury^43^,^44^,^45^. SDF-1 may play a role in the homing of microglia to areas of ischemic brain parenchyma. Interestingly, the homing of bone marrow-derived cells has been observed in the ischemic border between ischemic core and penumbra, similar to our results, and the expression of SDF-1 has been observed in the ischemic penumbra^44^. This ability of OGD-preconditioned microglia is crucial to the success of this cell-based therapy, although the mechanism by which OGD-preconditioned microglia reach the injured brain parenchyma remains to be elucidated.

Next, we demonstrated that optimal OGD preconditioning can induce anti-inflammatory M2 microglia, which are considered to have protective effects via the secretion of remodelling factors such as VEGF and TGF-β after cerebral ischemia^19^,^25^,^46^. Temporal analyses of microglial phenotypes in ischemic animals demonstrated that M2 microglia were detectable from 12 h, temporally increased at 1 to 3 days, and decreased after several days after cerebral ischemia^19^,^20^. In the present study, we chose 18 h incubation as the optimal OGD condition, because 18 h incubation induced predominantly M2 microglia whereas 24 h incubation caused cell death^33^,^34^. In fact, this OGD condition induced increased secretion of VEGF, MMP-9, and TGF-β *in vitro*, indicating the polarization of M2 microglia (Fig. 4). The administration of OGD-preconditioned microglia resulted in overexpression of remodelling factors such as VEGF, TGF-β, and MMP-9. These results showed the possible effects of OGD treatment on microglial M2 switch. The upregulation of these remodelling factors also was observed *in vivo,* more specifically in the injured brain parenchyma after OGD-preconditioned microglial transplantation.

We consider that the secretion of remodelling factors from OGD-preconditioned microglia were limited in duration because no GFP-positive OGD-preconditioned microglia were observed in the ischemic lesion at 21 days after transplantation. However, we observed the expression of these remodelling factors not only in the microglia but also in other cells at 28 days after transplantation, suggesting that changes in resident native endothelial cells, astrocytes, pericytes, neurons, and microglia might be caused by the paracrine action of OGD-preconditioned M2 microglia.

We speculate that pleiotropic effects of OGD-preconditioned M2 microglia, including the paracrine actions of the remodelling factors, and degradation of CSPG by MMP-9, might promote angiogenesis and axonal outgrowth (i.e., neuronal regeneration). We demonstrated that angiogenesis was activated at the border area within the MAP2-negative ischemic core, which we defined as the “angiogenesis-positive core” (Fig. 8B). In this region, we observed diminished expression of CSPG, one axonal outgrowth inhibitor^37^, which might be degraded by MMP-9^38^ (Fig. 8C). We also demonstrated that axonal outgrowth was observed only in the ischemic penumbra, especially around the region exhibiting angiogenesis. We consider that VEGF, MMP-9, and TGF-β might promote not only angiogenesis but also axonal outgrowth *in vivo* (Fig. 8B and C), because several studies have reported that these angiogenic factors are also involved in axonal outgrowth^23^,^47^,^48^. Interestingly, Lyden and others had proposed a ‘clean-up hypothesis’ whereby newborn vessels serve to facilitate macrophage/microglia infiltration and clear cellular debris from pan-necrotic tissue to facilitate angiogenesis and remodelling^49^,^50^. OGD-preconditioned microglia might induce this phenomenon in the “angiogenesis-positive core” and its surrounding tissue (i.e., penumbra) after cerebral ischemia. Based on these findings, we consider that cell-based therapies that cause a switch from M1- to M2-dominant microglia/macrophages might be promising.

In conclusion, we demonstrated that transplantation of microglia preconditioned by OGD may be a novel therapeutic strategy for ischemic stroke. We consider that the following therapeutic mechanisms might be involved: (i) migration of OGD-preconditioned microglia into the “angiogenesis-positive core”, (ii) secretion of remodelling factors from the M2 microglia stimulated by optimal OGD preconditioning, (iii) changes in resident native endothelial cells, astrocytes, pericytes, neurons, and microglia by paracrine actions of the M2 microglia, and (iv) angiogenesis and axonal outgrowth.

## Methods

This study was carried out in strict accordance with the recommendations from the Guide for the Care and Use of Laboratory Animals of the National Institutes of Health (Bethesda, MD, USA). The protocol was approved by the Niigata University Administrative Panel on Laboratory Animal Care. All surgeries were performed under inhalation of halothane and according to ARRIVE (Animal Research: Reporting of *In Vivo* Experiments) guidelines^51^. Rats and mice were maintained under controlled light (lights on, 5:00–19:00), temperature (23 ± 1 °C), and humidity (55 ± 10%) conditions and given free access to food and water.

### Focal cerebral ischemia

Transient focal cerebral ischemia was induced in male Sprague-Dawley rats weighing 290–320 g using an intraluminal filament suture technique^52^,^53^. After 90 min of ischemia, the suture was withdrawn to restore blood flow. This model provides an area of the ischemic core and penumbra determined by the presence of MAP2 with a high degree of reproducibility, and demonstrates that the time window for salvage of penumbral tissues by reperfusion was 90 min. Focal cerebral ischemia also was induced using a different model of focal embolic ischemia using an autologous thrombi technique^54^,^55^,^56^. Tissue plasminogen activator was administered intravenously in the form of Alteplase (Mitsubishi Tanabe Pharma Co., Osaka, Japan) at a dose of 10 mg/kg per animal at 4 h after cerebral ischemia for recanalization and reperfusion. Core body temperature, which was monitored via rectal probe, was maintained at 37.0 ± 0.5 °C using a heating pad.

### Experimental design

Sample size calculations were performed prior to the experiments to determine the number of animals needed to detect differences between cell-based therapy and control conditions. Based on a pilot study of N = 4 animals in the treatment group, we determined the sample size needed to detect differences in motor outcomes between the OGD-preconditioned microglial transplantation group and the control and normoxia groups (α, 0.05; one-sided analysis). Rats were randomly assigned to various experimental groups, and analyses were performed by an investigator blinded to the treatment.

### Immunofluorescence staining and confocal microscopy

The rats that survived for 1, 3, 7, 14, and 28 days after cerebral ischemia were euthanized with an overdose of halothane, followed by transcardial perfusion with cold normal saline followed by perfusion with cold 4% paraformaldehyde in 0.1 M phosphate-buffered saline (PBS; pH 7.4). Brains were removed and embedded in paraffin wax. Serial sections (4-μm thick) were cut from the paraffin blocks and stained using antibodies as described previously^53^. We also stained free-floating sections (50-μm thick)^53^. Sections were mounted with Vectashield 4′, 6′-diamidino-2-phenylindole (DAPI) (Vector Laboratories, Burlingame, CA, USA). Information about the primary antibodies is described in Supplementary Table 1. The sections were examined under a confocal laser-scanning microscope (LSM510 META; Carl Zeiss, Oberkochen, Germany). Cortical tissues corresponding to the ischemic core or penumbra were defined by MAP2 staining as described previously^52^,^53^.

### Immunocytochemistry

Microglial cells on coverslips were washed with PBS and fixed with 100% methanol for 10 seconds. After fixation, the cells were incubated with the primary fluorescein isothiocyanate (FITC)-conjugated anti-Mac-1 monoclonal antibody (Supplementary Table 1). Sections were mounted with DAPI (Vector Laboratories, Burlingame, CA, USA).

### Quantitative analysis of brain tissue structures and Mac-1 expression by immunostaining

To perform quantitative analyses of brain tissue structures, tissue sections were immunostained with antibodies against CD31 (a marker of endothelial cells and angiogenesis), MAP2 (a marker of neuronal dendrites), SMI31 and GAP43 (markers of neuronal axons), VEGF, TGF-β, and MMP-9 (Supplementary Table 1), and counted as described previously^55^. Briefly, seven randomly chosen non-overlapping high-power fields (630× or 1260×) at the level of the anterior commissure of the sham-operated or ischemic cortex in the MCA territory were examined. Data were acquired from stereotaxically identical 0.03-mm^3^ regions of interest (ROIs). Three-dimensional reconstructions and z-sections collected at 0.15-μm z-intervals were created and automatically quantified in blinded fashion as the intensity of total immunoreactive structure volumes (immunoreactive volume/examined ROI volume) using IMARIS imaging software (BitplaneAG, Zurich, Switzerland)^55^,^56^,^57^. The results were confirmed in three or four independent samples (N > 21–28).

For comparing the expression levels of Mac-1 in microglia under normoxic and OGD conditions, high-power fields (630×) were examined. Two-dimensional reconstructions were created and the intensity of total immunoreactivity per cell was quantified in a blinded manner using the IMARIS imaging software^34^ (N = 5).

### Cell counting protocol

To determine the frequency of cells positive for Ki67, a proliferative marker^39^, after cerebral ischemia, seven randomly chosen non-overlapping high-power fields (630×) at the level of the anterior commissure of the sham-operated or ischemic cortex (i.e., the border area of the ischemic core and penumbra) were examined at 1, 3, 7, and 14 days after cerebral ischemia (N = 21)^53^,^58^.

To determine the numbers of cells positive for M1 microglia-specific marker (iNOS) and M2-specific marker (CD206)^19^,^20^, seven randomly chosen non-overlapping high-power fields (630×) at the level of the anterior commissure of the ischemic cortex (i.e., the border area between the ischemic core and penumbra) were examined at 3 days after transplantation (10 days after cerebral ischemia (N > 21)^53^,^58^.

### Primary cell cultures

Primary murine microglia and astrocytes were obtained as previously described^33^. Primary mixed glial cultures were established from the forebrains of postnatal C57Bl/6 mice by dissociating isolated cerebral cortices in papain and then growing the resulting cell suspension for 10 days in Dulbecco’s modified Eagle’s medium (DMEM) supplemented with 10% foetal bovine serum (FBS). After 10 days, flasks were shaken for 15 min to remove loosely attached microglia. The purity of these microglial cultures was 99% as determined by Mac-1 (CD11b/CD18) immunoreactivity in flow cytometry^33^. For astrocytes, flasks were then shaken overnight to remove microglia and oligodendrocyte precursors. The remaining monolayer was determined as >95% astrocytes by glial fibrillary acidic protein (GFAP) immunoreactivity^33^.

### Oxygen–glucose deprivation

The standardized conditions for OGD have been described in detail elsewhere^33^,^35^. The cultures containing low-glucose medium were placed in a hypoxia chamber (Billups-Rothenburg, Del Mar, CA, USA), which was flushed with a mixture of 95% N_2_ and 5% CO_2_ for 1 h, and then closed for 18 h. Twenty-four h incubation under OGD conditions promoted cell death, whereas 18 h incubation under OGD conditions did not cause cell death as evaluated by propidium iodide^33^ and lactate dehydrogenase assays^34^. Additionally, M2 microglia were detectable starting from 12 h, maximally increasing at 24 h, and markedly decreased at later time points^20^. Given these findings, we chose 18 h for the OGD condition in this study.

### Enzyme-linked immunosorbent assay (ELISA)

After overnight incubation under OGD conditions, we measured the levels of VEGF, BDNF, TNF-α, TGF-β, IL-1β, IL-6, and IL-10 in the conditioned media using the VEGF Quantikine ELISA Kit (RRV00), BDNF Quantikine ELISA Kit (DBD00), TNF-α Quantikine ELISA Kit (MTA00B00), TGF-β Quantikine ELISA Kit (MB100B), IL-1β Quantikine ELISA Kit (MLB00C), IL-6 Quantikine ELISA Kit (M6000B), and IL-10 Quantikine ELISA Kit (M1000B) (all R&D Systems, Minneapolis, MN, USA), respectively, according to the manufacturer’s instructions (N = 5–6).

### Active MMP-9 assay

The Fluorimetric SensoLyte^TM^ 520 (AnaSpec Corp. San Jose, CA, USA) was used to quantify the specific enzymatic activity of active MMP-9 using a fluorescence resonance energy transfer (FRET) peptide containing a fluorescent donor and quenching acceptor^55^,^59^. We measured MMP-9 activities of the samples according to the manufacturer’s instructions. The protein concentrations of the samples were determined using a bicinchoninic acid protein assay kit (Pierce, Rockford, IL, USA). Samples (170 μg) were placed in a 96-well plate containing 50 μl of assay buffer. The proteolytic activities of MMP-9 were quantified using the FRET peptide.

### Cell transplantation

We excluded rats that weighed 2 SD below the mean at 7 days after cerebral ischemia to include only rats in the same physiological condition. Cells (1 × 10^6^, microglia or astrocytes) were diluted with 300 μL of PBS^13^. Rats subjected to transient MCA occlusion at 7 days after cerebral ischemia were randomly assigned to one of the cell-treated groups, in which transplantations of microglia or astrocytes were slowly infused through the stump of the external carotid artery over the course of 3 min (microglia group and astrocyte group, respectively), or the no cell control group. The same volume of PBS was injected in all groups.

### Sensorimotor assessment

Sensorimotor assessments were performed at 0, 1, 4, 7, 10 (3 days after transplantation), 14 (7 days after transplantation), 21 (14 days after transplantation), and 28 days (21 days after transplantation) after cerebral ischemia using the corner test^60^. Analyses of therapeutic effects were performed by an investigator blinded to treatment.

### Green fluorescent protein (GFP) mice

To determine whether transplanted microglia and astrocytes can translocate from the blood into the brain parenchyma to exert their beneficial effects after intra-arterial administration, we used primary microglia and astrocytes from GFP mice^30^. GFP transgenic mice were produced by breeding heterozygous pairs in the Genome Information Research Centre, Osaka University, Japan and maintained in the Department of Comparative and Experimental Medicine, Brain Research Institute, Niigata University. After preconditioning primary microglia and astrocytes from GFP mice by the OGD condition, we administered these cells intra-arterially at 7 days after cerebral ischemia. We performed confocal microscopic examination at 3 and 21 days after transplantation.

### Statistical analyses

All data are presented as the mean ± standard error of the mean (SEM). Differences in the parameters were analysed using one-way or two-way ANOVA followed by Bonferroni’s *post hoc* test or unpaired *t*-test. Statistical analyses were performed using IBM SPSS Statistics for Windows, Version 21.0 (Armonk, NY, USA). Differences in frequencies were assessed with Fisher’s exact test. All tests were considered statistically significant at a P value < 0.05.

## Additional Information

**How to cite this article**: Kanazawa, M. *et al*. Microglia preconditioned by oxygen-glucose deprivation promote functional recovery in ischemic rats. *Sci. Rep.* **7**, 42582; doi: 10.1038/srep42582 (2017).

**Publisher's note:** Springer Nature remains neutral with regard to jurisdictional claims in published maps and institutional affiliations.

## Supplementary Material

Supplementary Information

## References

[b1] Mozaffarian D. . Heart disease and stroke statistics–2015 update: a report from the American Heart Association. Circulation 131, e29–322 (2015). 25520374 10.1161/CIR.0000000000000152

[b2] Winstein C. J. . Guidelines for Adult Stroke Rehabilitation and Recovery: A Guideline for Healthcare Professionals From the American Heart Association/American Stroke Association. Stroke 47, e98–169 (2016). 27145936 10.1161/STR.0000000000000098

[b3] Freytes D. O. . Macrophages modulate the viability and growth of human mesenchymal stem cells. J. Cell. Biochem. 114, 220–229 (2013). 22903635 10.1002/jcb.24357

[b4] Arai K. . Brain angiogenesis in developmental and pathological processes: neurovascular injury and angiogenic recovery after stroke. FEBS J 276, 4644–4652 (2009). 19664070 10.1111/j.1742-4658.2009.07176.x PMC3712842

[b5] Zhang Z. G. . VEGF enhances angiogenesis and promotes blood-brain barrier leakage in the ischemic brain. J. Clin. Invest. 106, 829–838 (2000). 11018070 10.1172/JCI9369 PMC517814

[b6] Hariawala M. D. . VEGF improves myocardial blood flow but produces EDRF-mediated hypotension in porcine hearts. J. Surg. Res. 63, 77–82 (1996). 8661176 10.1006/jsre.1996.0226

[b7] Yang R. . Effects of the vascular endothelial growth factor on hemodynamics and cardiac performance. J. Cardiovasc. Pharmacol. 27, 838–844 (1996). 8761851 10.1097/00005344-199606000-00011

[b8] Yang R. . Substantially attenuated hemodynamic responses to Escherichia coli-derived vascular endothelial growth factor given by intravenous infusion compared with bolus injection. J. Pharmacol. Exp. Ther. 284, 103–110 (1998). 9435167

[b9] Moskowitz M. A. . The science of stroke: mechanisms in search of treatments. Neuron 67, 181–198 (2010). 20670828 10.1016/j.neuron.2010.07.002 PMC2957363

[b10] Liu X. . Cell based therapies for ischemic stroke: from basic science to bedside. Prog. Neurobiol. 115, 92–115 (2014). 24333397 10.1016/j.pneurobio.2013.11.007 PMC4038267

[b11] Li Q. . Modeling the neurovascular niche: VEGF- and BDNF-mediated cross-talk between neural stem cells and endothelial cells: an *in vitro* study. J. Neurosci. Res. 84, 1656–1668 (2006). 17061253 10.1002/jnr.21087

[b12] Moriyama Y. . Intravenous injection of neural progenitor cells facilitates angiogenesis after cerebral ischemia. Brain Behav. 3, 43–53 (2013). 23532762 10.1002/brb3.113 PMC3607146

[b13] Ishizaka S. . Intra-arterial cell transplantation provides timing-dependent cell distribution and functional recovery after stroke. Stroke 44, 720–726 (2013). 23362081 10.1161/STROKEAHA.112.677328

[b14] Andres R. H. . Human neural stem cells enhance structural plasticity and axonal transport in the ischaemic brain. Brain 134, 1777–1789 (2011). 21616972 10.1093/brain/awr094 PMC3102243

[b15] Prasad K. . Intravenous autologous bone marrow mononuclear stem cell therapy for ischemic stroke: a multicentric, randomized trial. Stroke 45, 3618–3624 (2014). 25378424 10.1161/STROKEAHA.114.007028

[b16] Seto T. . Brain magnetic resonance imaging in 23 patients with mucopolysaccharidoses and the effect of bone marrow transplantation. Ann. Neurol. 50, 79–92 (2001). 11456314 10.1002/ana.1098

[b17] Iadecola C. & Anrather J. The immunology of stroke: from mechanisms to translation. Nat. Med. 17, 796–808 (2011). 21738161 10.1038/nm.2399 PMC3137275

[b18] Mabuchi T. . Contribution of microglia/macrophages to expansion of infarction and response of oligodendrocytes after focal cerebral ischemia in rats. Stroke 31, 1735–1743 (2000). 10884481 10.1161/01.str.31.7.1735

[b19] Hu X. . Microglia/macrophage polarization dynamics reveal novel mechanism of injury expansion after focal cerebral ischemia. Stroke 43, 3063–3070 (2012). 22933588 10.1161/STROKEAHA.112.659656

[b20] Perego C. . Temporal pattern of expression and colocalization of microglia/macrophage phenotype markers following brain ischemic injury in mice. J. Neuroinflammation 8, 174 (2011). 22152337 10.1186/1742-2094-8-174 PMC3251548

[b21] Crain J. M. . Microglia express distinct M1 and M2 phenotypic markers in the postnatal and adult central nervous system in male and female mice. J. Neurosci. Res. 91, 1143–1151 (2013). 23686747 10.1002/jnr.23242 PMC3715560

[b22] Imai F. . Neuroprotective effect of exogenous microglia in global brain ischemia. J. Cereb. Blood Flow Metab. 27, 488–500 (2007). 16820801 10.1038/sj.jcbfm.9600362

[b23] Rosenberg G. A. Matrix metalloproteinases and their multiple roles in neurodegenerative diseases. Lancet Neurol. 8, 205–216 (2009). 19161911 10.1016/S1474-4422(09)70016-X

[b24] Vivien D. & Ali C. Transforming growth factor-beta signalling in brain disorders. Cytokine Growth Factor Rev. 17, 121–128 (2006). 16271500 10.1016/j.cytogfr.2005.09.011

[b25] Stankovic N. D. . Microglia-blood vessel interactions: a double-edged sword in brain pathologies. Acta. Neuropathol. 131, 347–363 (2016). 26711460 10.1007/s00401-015-1524-y

[b26] Hughes P. M. . Monocyte chemoattractant protein-1 deficiency is protective in a murine stroke model. J. Cereb. Blood Flow Metab. 22, 308–317 (2002). 11891436 10.1097/00004647-200203000-00008

[b27] Neumann H. Microglia: a cellular vehicle for CNS gene therapy. J. Clin. Invest. 116, 2857–2860 (2006). 17080190 10.1172/JCI30230 PMC1626126

[b28] Wattananit S. . Monocyte-derived macrophages contribute to spontaneous long-term functional recovery after stroke in mice. J. Neurosci. 36, 4182–4195 (2016). 27076418 10.1523/JNEUROSCI.4317-15.2016 PMC6601783

[b29] Ueno Y. . Axonal outgrowth and dendritic plasticity in the cortical peri-infarct area after experimental stroke. Stroke 43, 2221–2228 (2012). 22618383 10.1161/STROKEAHA.111.646224 PMC3404219

[b30] Okabe M. . Green mice’ as a source of ubiquitous green cells. FEBS Lett. 407, 313–319 (1997). 9175875 10.1016/s0014-5793(97)00313-x

[b31] Soriano S. . Mice deficient in Mac-1 (CD11b/CD18) are less susceptible to cerebral ischemia/reperfusion injury. Stroke 30, 134–139 (1999). 9880401 10.1161/01.str.30.1.134

[b32] Ahn G. O. . Inhibition of Mac-1 (CD11b/CD18) enhances tumor response to radiation by reducing myeloid cell recruitment. Proc. Natl. Acad. Sci. USA 107, 8363–8368 (2010). 20404138 10.1073/pnas.0911378107 PMC2889597

[b33] Milner R. . Responses of endothelial cell and astrocyte matrix-integrin receptors to ischemia mimic those observed in the neurovascular unit. Stroke 39, 191–197 (2008). 18032737 10.1161/STROKEAHA.107.486134 PMC2588548

[b34] Kanazawa M. . Multiple therapeutic effects of progranulin on experimental acute ischaemic stroke. Brain 138, 1932–1948 (2015). 25838514 10.1093/brain/awv079

[b35] del Zoppo G. J. . Microglial cell activation is a source of metalloproteinase generation during hemorrhagic transformation. J. Cereb. Blood Flow Metab. 32, 919–932 (2012). 22354151 10.1038/jcbfm.2012.11 PMC3345906

[b36] Arai K. . Essential role for ERK mitogen-activated protein kinase in matrix metalloproteinase-9 regulation in rat cortical astrocytes. Glia 43, 254–264 (2003). 12898704 10.1002/glia.10255

[b37] Galtrey C. M. & Fawcett J. W. The role of chondroitin sulfate proteoglycans in regeneration and plasticity in the central nervous system. Brain Res. Rev. 54, 1–18 (2007). 17222456 10.1016/j.brainresrev.2006.09.006

[b38] Larsen P. H. . Matrix metalloproteinase-9 facilitates remyelination in part by processing the inhibitory NG2 proteoglycan. J. Neurosci. 23, 11127–11135 (2003). 14657171 10.1523/JNEUROSCI.23-35-11127.2003 PMC6741053

[b39] Wang Z. . Chronic valproate treatment enhances postischemic angiogenesis and promotes functional recovery in a rat model of ischemic stroke. Stroke 43, 2430–2436 (2012). 22811460 10.1161/STROKEAHA.112.652545 PMC3429729

[b40] Dawson D. A. . Acute focal ischemia-induced alterations in MAP2 immunostaining: description of temporal changes and utilization as a marker for volumetric assessment of acute brain injury. J. Cereb. Blood Flow Metab. 16, 170–174 (1996). 8530550 10.1097/00004647-199601000-00020

[b41] Zhao H. . Akt contributes to neuroprotection by hypothermia against cerebral ischemia in rats. J. Neurosci. 25, 9794–9806 (2005). 16237183 10.1523/JNEUROSCI.3163-05.2005 PMC6725740

[b42] Li Y. . Neuronal damage and plasticity identified by microtubule-associated protein 2, growth-associated protein 43, and cyclin D1 immunoreactivity after focal cerebral ischemia in rats. Stroke 29, 1972–1980 (1998). 9731626 10.1161/01.str.29.9.1972

[b43] Lu D. Y. . SDF-1alpha up-regulates interleukin-6 through CXCR4, PI3K/Akt, ERK, and NF-kappaB-dependent pathway in microglia. Eur. J. Pharmacol. 613, 146–154 (2009). 19285061 10.1016/j.ejphar.2009.03.001

[b44] Hill W. . SDF-1 (CXCL12) is upregulated in the ischemic penumbra following stroke: association with bone marrow cell homing to injury. J. Neuropathol. Exp. Neurol. 63, 84–96 (2004). 14748564 10.1093/jnen/63.1.84

[b45] Robin A. . Stromal cell-derived factor 1a mediates neural progenitor cell motility after focal cerebral ischemia. J. Cereb. Blood Flow Metab. 26, 125–134 (2006) 15959456 10.1038/sj.jcbfm.9600172

[b46] Cherry J. D. . Neuroinflammation and M2 microglia: the good, the bad, and the inflamed. J. Neuroinflammation 11, 98 (2014). 24889886 10.1186/1742-2094-11-98 PMC4060849

[b47] Sondell M. . Vascular endothelial growth factor has neurotrophic activity and stimulates axonal outgrowth, enhancing cell survival and Schwann cell proliferation in the peripheral nervous system. J. Neurosci. 19, 5731–5740 (1999). 10407014 10.1523/JNEUROSCI.19-14-05731.1999 PMC6783109

[b48] Ho T. W. . TGFβ trophic factors differentially modulate motor axon outgrowth and protection from excitotoxicity. Exp. Neurol. 161, 664–675 (2000). 10686085 10.1006/exnr.1999.7290

[b49] Manoonkitiwongsa P. S. . Angiogenesis after stroke is correlated with increased numbers of macrophages: the clean-up hypothesis. J. Cereb. Blood Flow Metab. 21, 1223–1231 (2001). 11598500 10.1097/00004647-200110000-00011

[b50] Yu S. W. . Stroke-evoked angiogenesis results in a transient population of microvessels. J. Cereb. Blood Flow Metab. 27, 755–763 (2007). 16883352 10.1038/sj.jcbfm.9600378

[b51] Kilkenny C. . National Centre for the Replacement, Refinement and Reduction of Amimals in Research. Animal research: reporting *in vivo* experiments—the ARRIVE guidelines. J. Cereb. Blood Flow Metab. 31, 991–993 (2010). 10.1038/jcbfm.2010.220 PMC3070981 21206507

[b52] Memezawa H. . Penumbral tissues salvaged by reperfusion following middle cerebral artery occlusion in rats. Stroke 23, 552–559 (1992). 1561688 10.1161/01.str.23.4.552

[b53] Kanazawa M. . Biochemical and histopathological alterations in TAR DNA binding protein-43 after acute ischemic stroke in rats. J. Neurochem. 116, 957–965 (2011). 20557425 10.1111/j.1471-4159.2010.06860.x

[b54] Okubo S. . FK-506 extended the therapeutic time window for thrombolysis without increasing the risk of hemorrhagic transformation in an embolic rat stroke model. Brain Res. 1143, 221–227 (2007). 17316578 10.1016/j.brainres.2007.01.050

[b55] Kanazawa M. . Inhibition of VEGF signaling pathway attenuates hemorrhage after tPA treatment. J. Cereb. Blood Flow Metab. 31, 1461–1474 (2011). 21304556 10.1038/jcbfm.2011.9 PMC3130331

[b56] Kawamura K. . Effects of angiopoietin-1 on hemorrhagic transformation and cerebral edema after tissue plasminogen activator treatment for ischemic stroke in rats. PLoS One 9, e98639 (2014). 24896569 10.1371/journal.pone.0098639 PMC4045756

[b57] Zhang L. . Atorvastatin extends the therapeutic window for tPA to 6 h after the onset of embolic stroke in rats. J. Cereb. Blood Flow Metab. 29, 1816–1824 (2009). 19638998 10.1038/jcbfm.2009.105 PMC2845317

[b58] Katchanov J. . Mild cerebral ischemia induces loss of cyclin-dependent kinase inhibitors and activation of cell cycle machinery before delayed neuronal cell death. J. Neurosci. 21, 5045–5053 (2001). 11438580 10.1523/JNEUROSCI.21-14-05045.2001 PMC6762829

[b59] Lin C. W. . Quercetin inhibition of tumor invasion via suppressing PKCδ/ERK/AP-1-dependent matrix metalloproteinase-9 activation in breast carcinoma cells. Carcinogenesis 29, 1807–1815 (2008). 18628248 10.1093/carcin/bgn162

[b60] Balkaya M. . Characterization of long-term functional outcome in a murine model of mild brain ischemia. J. Neurosci. Methods 213, 179–187 (2013). 23291083 10.1016/j.jneumeth.2012.12.021

