# Supplementary material for: Microglia preconditioned by oxygen-glucose deprivation promote functional recovery in ischemic rats
Source: Sci Rep. 2017 Feb 14;7:42582. doi: 10.1038/srep42582 (PMC5307390; doi:10.1038/srep42582)
Supplement: Supplementary Information [file srep42582-s1.pdf]

Microglia preconditioned by oxygen-glucose deprivation promote functional recovery in ischemic rats

Masato Kanazawa, M.D., Ph.D.,[1] Minami Miura, M.Med.,[1] Masafumi Toriyabe, M.D., Ph.D.[1] Misaki Koyama, M.Med,[1] Masahiro Hatakeyama, M.D.,[1] Masanori Ishikawa, M.D., Ph.D.,[1] Takashi Nakajima, M.D., Ph.D.,[2] Osamu Onodera, M.D., Ph.D.,[1] Tetsuya Takahashi, M.D., Ph.D.,[1] Masatoyo Nishizawa, M.D., Ph.D.,[1] and Takayoshi Shimohata, M.D., Ph.D.[1]

[1]Department of Neurology, Brain Research Institute, Niigata University, 1-757 Asahimachi-dori, Chuoku, Niigata, Niigata, Japan

[2]Department of Neurology, Niigata National Hospital, National Hospital Organization, 3-52 Akasaka-cho, Kashiwazaki, Niigata, Japan

Supplementary Table 1

Antibodies	Source	Dilutions	Manufacturer	Catalog #
CD31	Rat	1:20	Dianova	DIA-310
CD68/ED1	Mouse	1:500	BMA Biomedicals	T-3003
CD206	Gout	1:500	R&D Systems, Inc	AF2523
CSPG (NG2)	Mouse	1:200	Millipore	#05-710
GAP43	Mouse	1:500	NOVUS Biochemicals	NB300-143
GFAP	Mouse	1:50	Cell Signaling Technology	#3670
iNOS	Rabbit	1:300	Abcam	ab15323
Ki67	Rabbit	1:1000	Abcam	ab15580
Mac-1 (CD11b/CD18)	Rat	1:100	Abcam	ab24874
MAP2	Mouse	1:250	Sigma-Aldrich	M9942
MMP-9	Rabbit	1:200	Millipore	ab19016
PDGFRβ	Gout	1:500	R&D Systems, Inc	AF1042
SMI31	Mouse	1:500	Convance	SMI-31R
TGF-β	Rabbit	1:500	Torrey Prince Biolabs	TP254
VEGF	Rabbit	1:200	Santa Cruz Biotechnologies	sc-152

Supplementary Table 1. Information of primary antibodies used in the present study

CD11b, cluster of differentiation 11b; CD18, cluster of differentiation 18; CD31, cluster of differentiation 31; CD68, cluster of differentiation 68; CD206, cluster of differentiation 206; CSPG, chondroitin sulphate proteoglycan; GAP43, growth associated protein 43; GFAP, glial fibrillary acidic protein; iNOS, inducible nitric oxide synthase; Mac-1, macrophage-1; MAP2, microtubule-associated protein 2; MMP-9, matrix metalloproteinase-9; NG2, neuron-glial antigen 2; PDGFRβ, platelet-derived growth factor receptor-β; TGF-β, transforming growth factor-β; VEGF, vascular endothelial growth factor.

Supplementary Figure 1

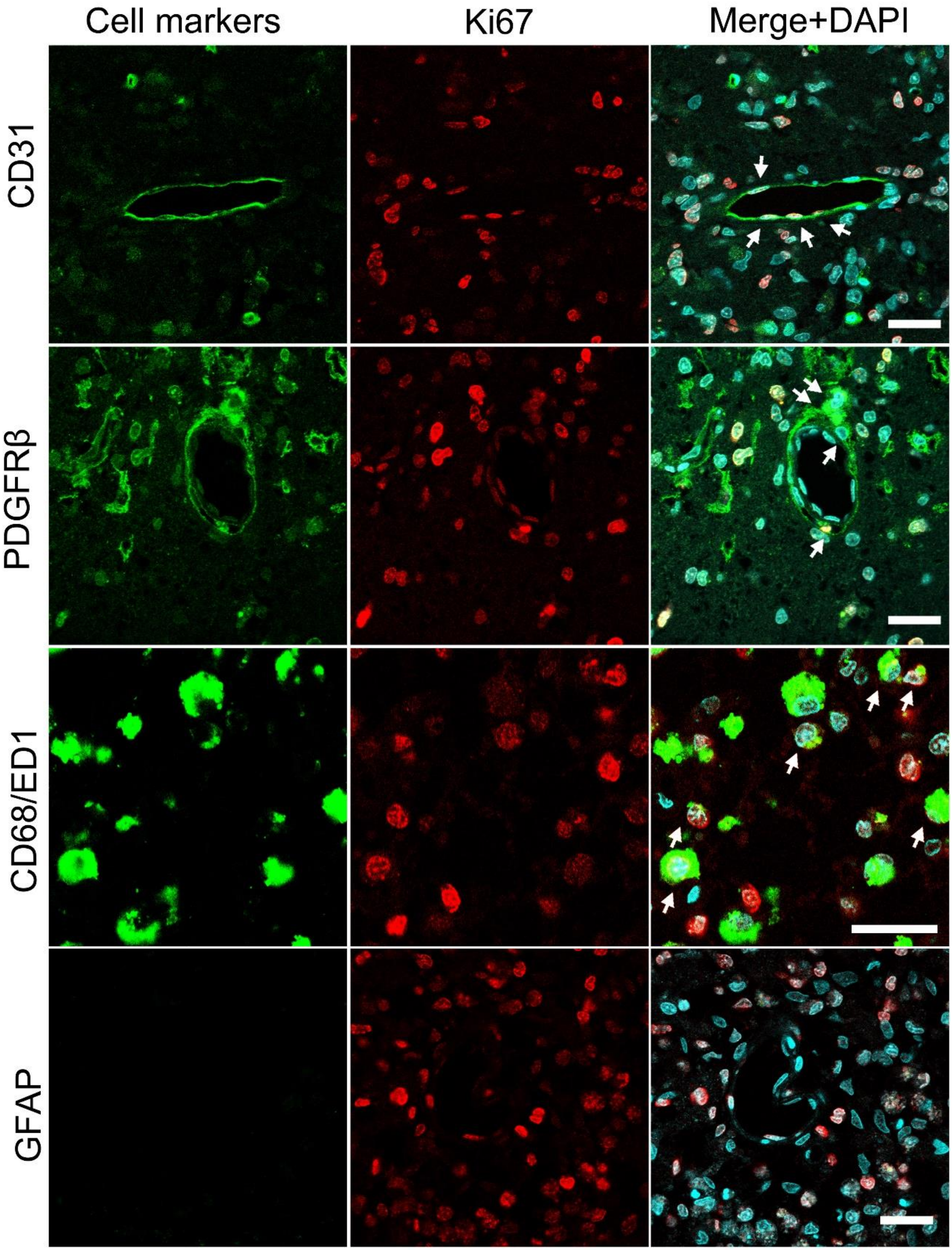

Supplementary Figure 2

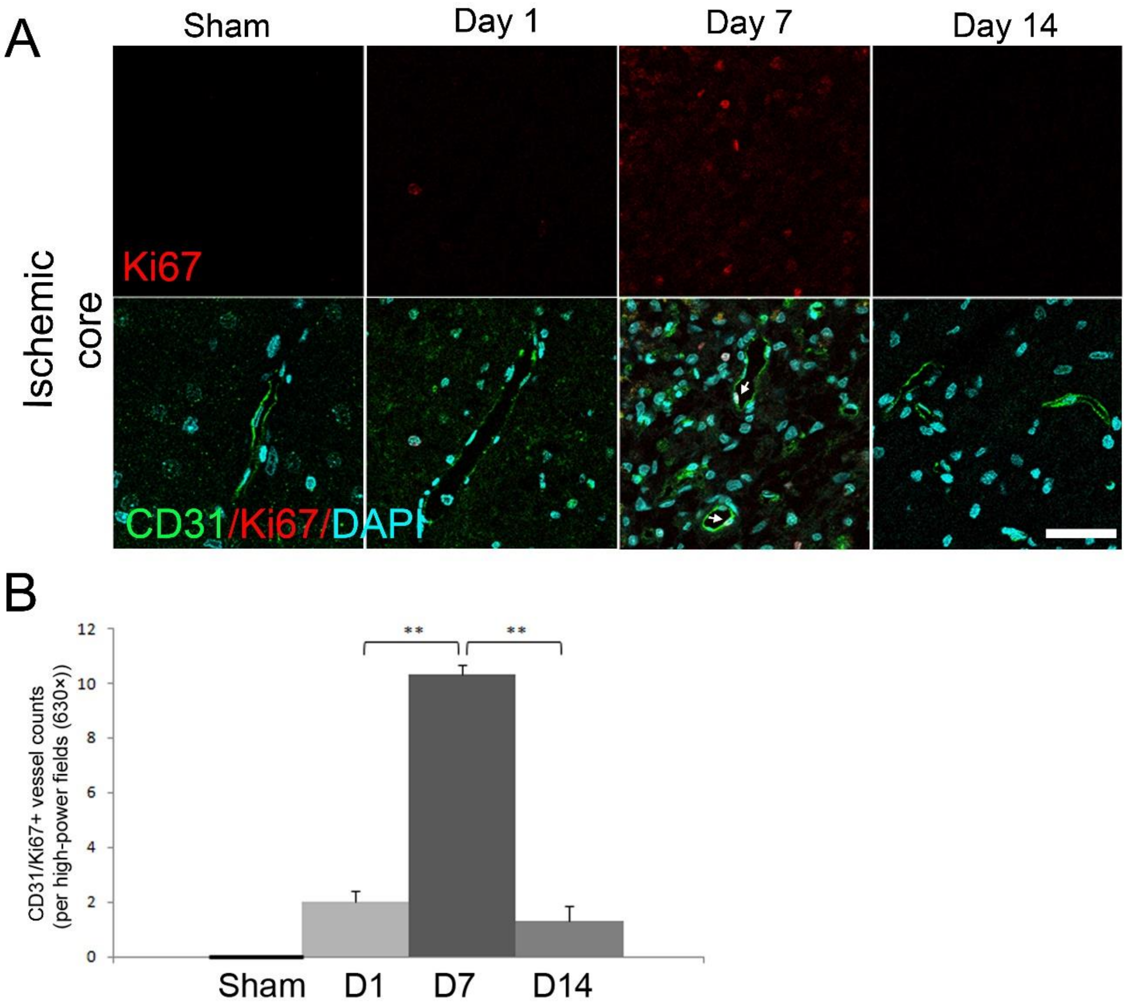

Supplementary Figure 3

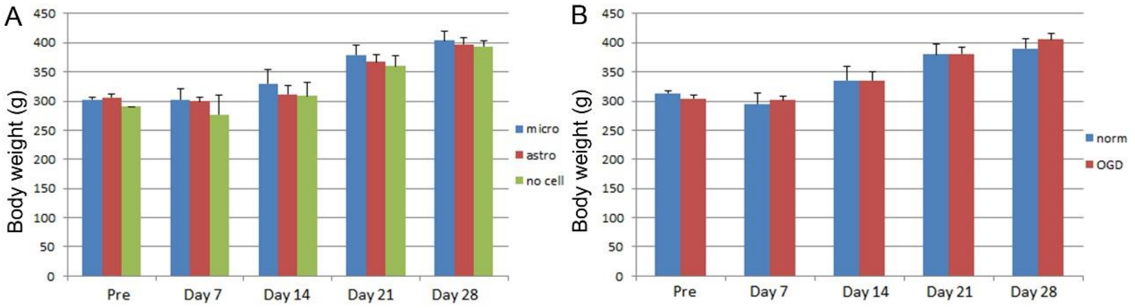

Supplementary Figure 4

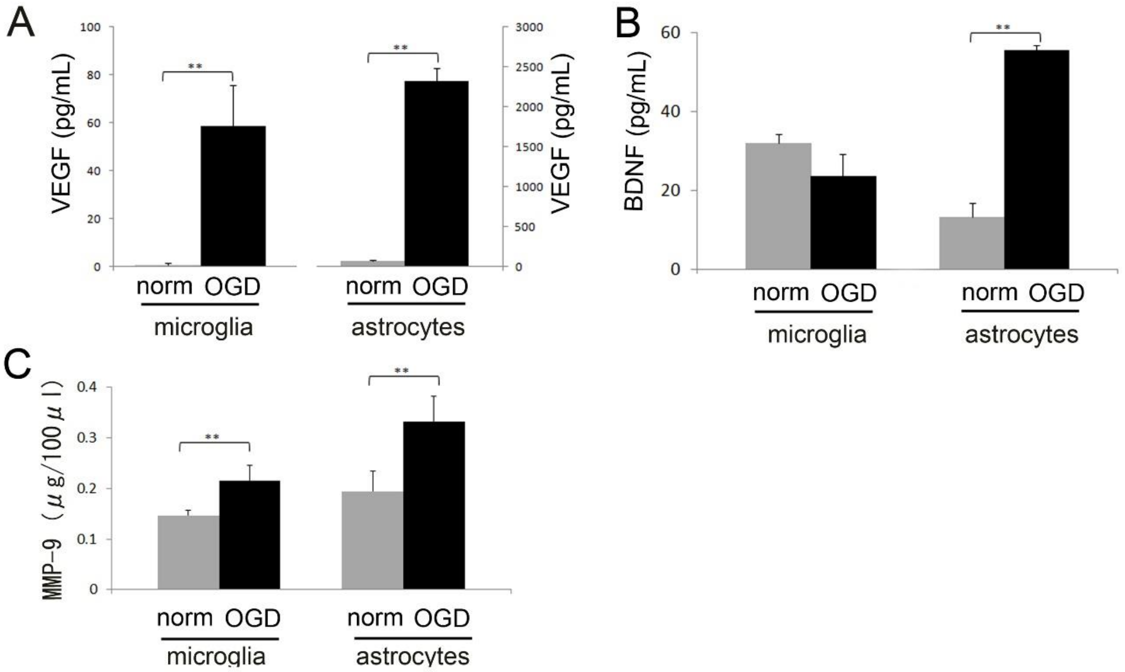

Supplementary Figure 5

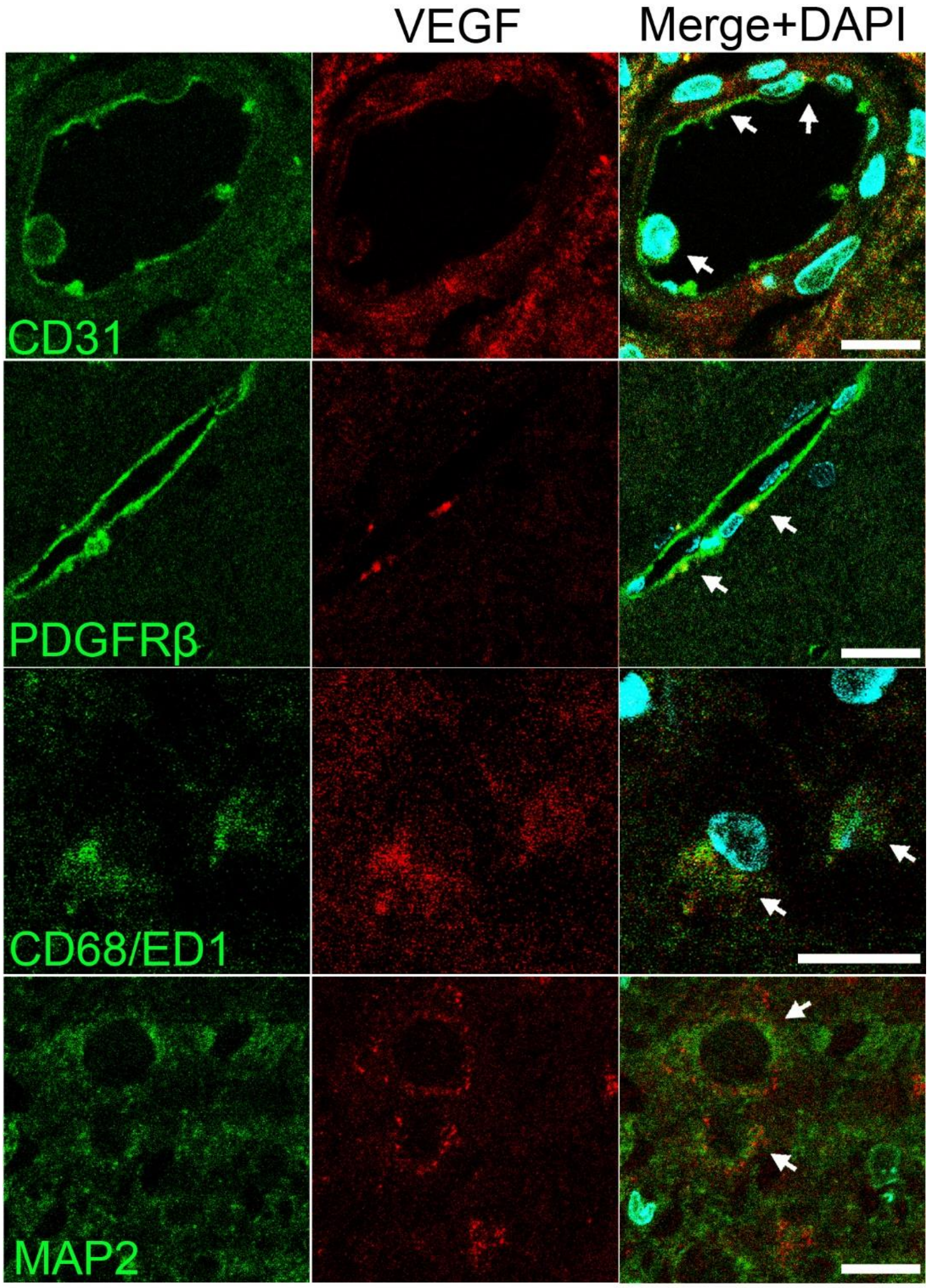

Supplementary Figure 6

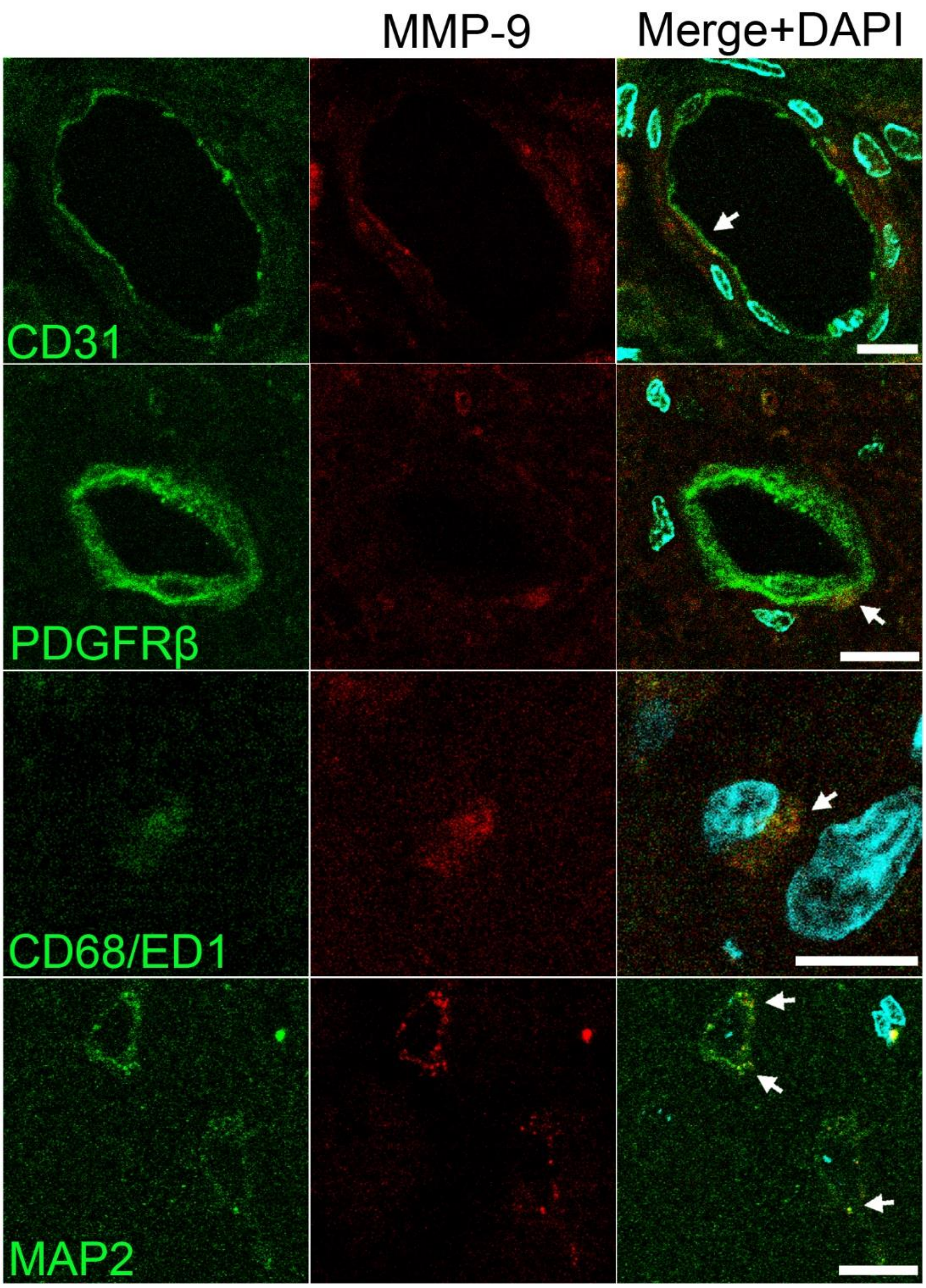

Supplementary Figure 7

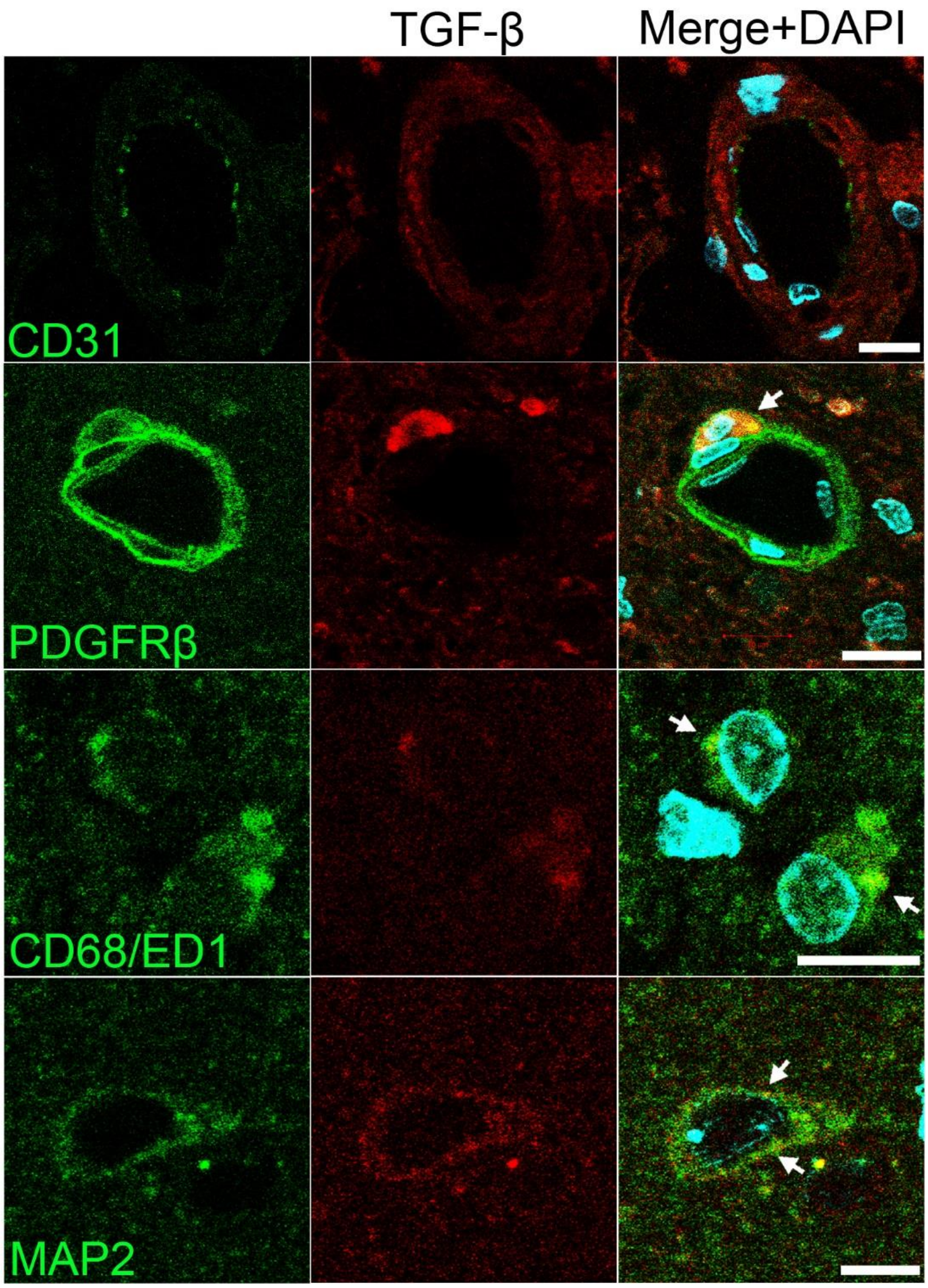

Supplementary Figure 8

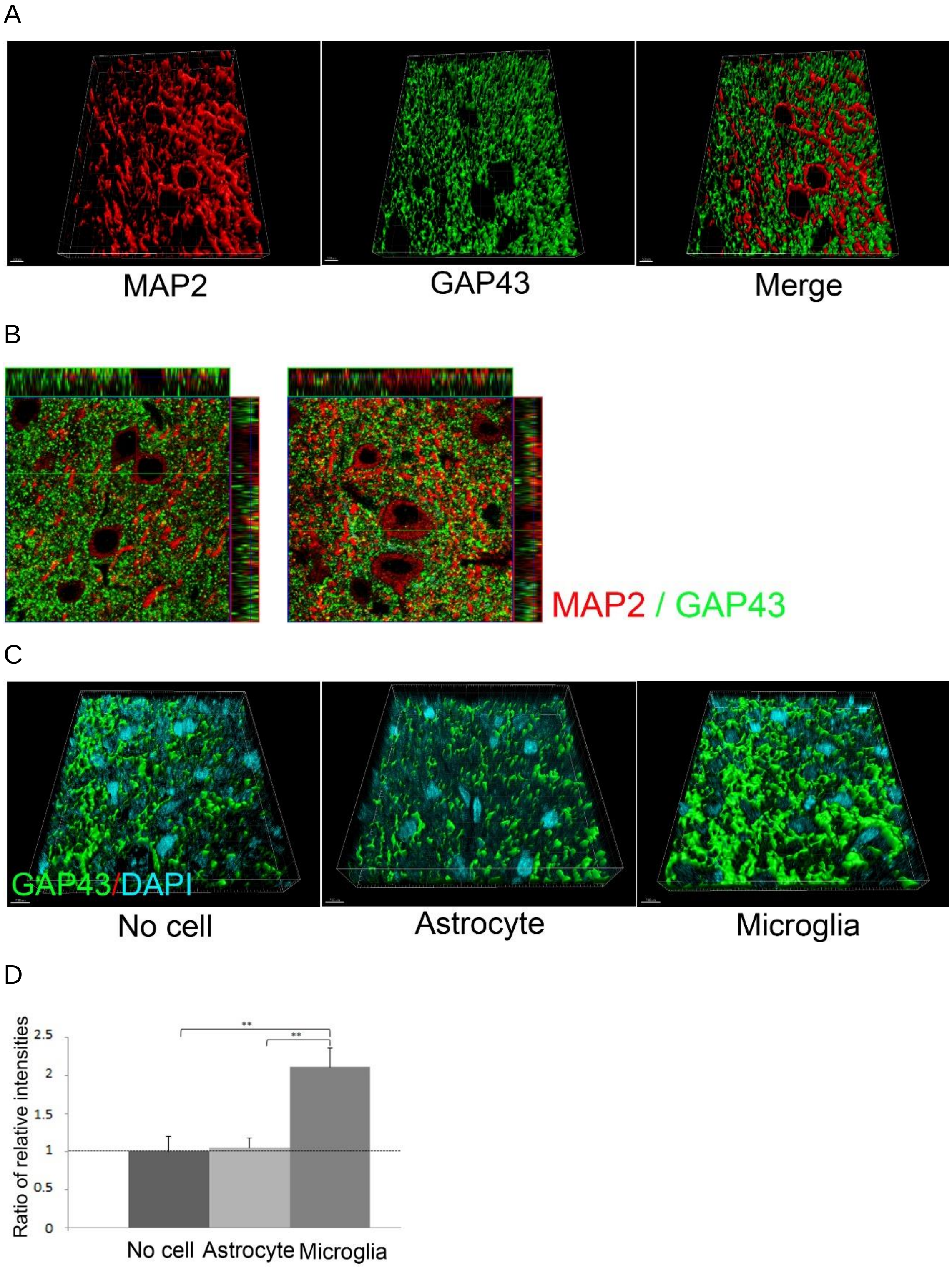

Supplementary Figure Legends:

Supplementary Figure 1. Cellular expressions of cell proliferation marker Ki67 in ischemic rats.

Triple staining for cluster of differentiation 31 (CD31, a marker of endothelial cells; green), platelet-derived growth factor receptor-β (PDGFRβ, a marker of pericytes; green), CD68/ED1 (a marker of microglia; green), or glial fibrillary acidic protein (GFAP, a marker of astrocytes; green); Ki67 (red); and 4',6'-diamidino-2-phenylindole (DAPI; blue) was performed using ischemic rat brains at 7 days after cerebral ischemia. Arrows indicate expression of Ki67 in endothelial cells (top row), pericytes (second row), and microglia (third row), but not in astrocytes (bottom row) in rats after cerebral ischemia. Scale bars, 20 μm.

Supplementary Figure 2. Temporal changes in angiogenesis in rat cerebral cortex after thromboembolic cerebral ischemia.

(A) Confocal microscopic images of CD31 (green)/Ki67 (red)/DAPI (blue) triple labelling of cerebral cortices in the non-ischemic (sham-operated) and ischemic core at 1, 7, and 14 days after cerebral ischemia in the thromboembolic model. Arrows indicate Ki67-positive angiogenic endothelial nuclei. Scale bars, 20 μm. **(B)** Number of CD31/Ki67 double-positive vessels from the ischemic core at 1 (D1), 7 (D7), and 14

(D14) days after cerebral ischemia (N = 21). **P < 0.01.

Supplementary Figure 3. Body weight changes in ischemic rats after cell therapy.

(A) Body weights in rats from the oxygen-glucose deprivation (OGD)-preconditioned microglia transplanted group (micro), OGD-preconditioned astrocyte transplanted group (astro), and no cell control group (no cell) examined at 0, 7, 14, 21, and 28 days after cerebral ischemia (N = 4). **(B)** Body weights in rats from the normoxic microglia transplanted group (norm) and OGD-preconditioned microglia transplanted group (OGD) examined at 0, 7, 14, 21, and 28 days after cerebral ischemia (N = 6).

Supplementary Figure 4. Characteristics of murine primary microglia and astrocytes subjected to normoxic or oxygen-glucose deprivation (OGD) conditions.

The levels of secretory vascular endothelial growth factor (VEGF) (**A**), brain-derived neurotrophic factor (BDNF) **(B),** and matrix metalloproteinase-9 (MMP-9) **(C)** from conditioned media of murine primary cultured microglia and astrocytes subjected to normoxia (norm) or OGD condition (N = 6-8 each). **P < 0.01.

Supplementary Figure 5. Cellular expression of vascular endothelial growth factor (VEGF) in ischemic rats.

VEGF was expressed in endothelial cells (top row), pericytes (second row), microglia (third row), and neurons (bottom row) in rats after cerebral ischemia. Triple staining for cluster of differentiation 31 (CD31, a marker of endothelial cells; green), platelet-derived growth factor receptor-β (PDGFRβ, a marker of pericytes; green), CD68/ED1 (a marker of microglia; green), or microtubule-associated protein 2 (MAP2, a marker of neurons; green); VEGF (red); and 4’, 6’-diamidino-2-phenylindole (DAPI; blue) was performed using ischemic rat brains at 28 days after cerebral ischemia. Arrows indicate VEGF expression. Scale bars, 10 μm.

Supplementary Figure 6. Cellular expression of matrix metalloproteinase-9 (MMP-9) in ischemic rats.

MMP-9 was expressed in endothelial cells (top row), pericytes (second row), microglia (third row), and neurons (bottom row) in rats after cerebral ischemia. Triple staining for cluster of differentiation 31 (CD31, a marker of endothelial cells; green), platelet-derived growth factor receptor-β (PDGFRβ, a marker of pericytes; green), CD68/ED1 (a marker of microglia; green), or microtubule-associated protein 2 (MAP2, a marker of neurons; green); MMP-9 (red); and 4’, 6’-diamidino-2-phenylindole (DAPI; blue) was performed using ischemic rat brains at 28 days after cerebral ischemia. Arrows indicate MMP-9 expression. Scale bars, 10 μm.

Supplementary Figure 7. Cellular expression of transforming growth factor-β (TGF-β) among ischemic rats.

TGF-β was expressed in pericytes (second row), microglia (third row), and neurons (bottom row), but not in endothelial cells (top row) in rats after cerebral ischemia. Triple staining for cluster of differentiation 31 (CD31, a marker of endothelial cells; green), platelet-derived growth factor receptor-β (PDGFRβ, a marker of pericytes; green), CD68/ED1 (a marker of microglia; green), or microtubule-associated protein 2 (MAP2, a marker of neurons; green); TGF-β (red); and 4’, 6’-diamidino-2-phenylindole (DAPI; blue) was performed using ischemic rat brains at 28 days after cerebral ischemia. Arrows indicate TGF-β expression. Scale bars, 10 μm.

Supplementary Figure 8. Transplantation of oxygen-glucose deprivation (OGD)-preconditioned microglia promotes axonal outgrowth in the ischemic penumbra at 28 days after cerebral ischemia.

Representative figures of microtubule-associated protein 2 (MAP2) and growth associated protein 43 (GAP43) from cerebral cortices of sham-operated rats (**A, B**). MAP2 (red)/GAP43 (green) double labelling in ischemic cortices at 28 days after cerebral ischemia as examined by confocal microscopy. Three-dimensional (**A**) and two-

dimensional images (**B**) revealed that MAP2 did not colocalise with GAP43. Representative figures (**C**) and the ratio of relative signal intensities of GAP43 (**D**) in the ischemic penumbra from the cerebral cortices of the OGD-preconditioned microglia or OGD-preconditioned astrocyte transplanted groups and no cell treatment group at 28 days after cerebral ischemia. GAP43 (green)/4', 6'-diamidino-2-phenylindole (DAPI; blue) double labelling in the ischemic penumbra at 28 days after cerebral ischemia as examined by confocal microscopy. Scale bars, 15 μm. The bar graph represents the relative signal intensities of the OGD-preconditioned microglia or astrocyte transplanted ischemic brain samples compared with those of no cell control samples (N = 21-28). **P < 0.01.
